# Circulating low- and medium-molecular-weight adiponectin are associated with metabolic phenotypes in adults undergoing cardiometabolic screening

**DOI:** 10.1371/journal.pone.0360041

**Published:** 2026-10-05

**Authors:** Sergio Martínez-Huenchullán, Nicole Cares, Josefina Enríquez, Consuelo Quezada, Jessica Molina, Teresa Ross, Amanda Bentes, Yanara Pavez, Paola Rubilar, Catalina Ramírez-Contreras, Monserrat Fitó, Álvaro Hernáez, Viviana Sandoval

**Affiliations:** 1 Carrera de Kinesiología, Facultad de Ciencias de la Rehabilitación y Calidad de Vida, Universidad San Sebastián, Valdivia, Chile; 2 Centro de Biología Celular y Biomedicina (CEBICEM), Facultad de Ciencias, Universidad San Sebastián, Santiago, Chile; 3 Carrera de Tecnología Médica, Facultad de Ciencias, Universidad San Sebastián, Valdivia, Chile; 4 Carrera de Nutrición y Dietética, Facultad de Ciencias de la Rehabilitación y Calidad de Vida, Universidad San Sebastián, Valdivia, Chile; 5 Instituto de Ciencias de la Educación, Universidad Austral de Chile, Valdivia, Chile; 6 Facultad de Medicina, Instituto de Anatomía, Patología e Histología, Universidad Austral de Chile, Valdivia, Chile; 7 Doctorado en Enfermedades Crónicas, Facultad de Ciencias, Universidad San Sebastián, Santiago, Chile; 8 Facultad de Medicina y Ciencias de la Salud, Universidad Central de Chile, Carrera de Tecnología Médica, Santiago, Chile; 9 Departamento de Nutrición y Salud Pública, Facultad de Ciencias de la Salud y los Alimentos, Universidad del Bío-Bío, Chillán, Chile; 10 Hospital del Mar Research Institute (HMRIB), Barcelona, Spain; 11 CIBER de Fisiopatología de la Obesidad y Nutrición (CIBEROBN), Instituto de Salud Carlos III, Madrid, Spain; 12 Universitat Ramon Llull, Blanquerna School of Health Sciences, Barcelona, Spain; 13 CIBER de Enfermedades Cardiovasculares (CIBERCV), Instituto de Salud Carlos III, Madrid, Spain; Waseda University: Waseda Daigaku, JAPAN

## Abstract

**Background and aims:**

Adiponectin circulates as multimers with potentially distinct biological roles, yet most human studies report total adiponectin, which may mask multimer-specific metabolic information**.** This study aimed to determine whether circulating low- (LMW) and medium-molecular-weight (MMW) adiponectin and their relative pattern are associated with cardiometabolic, behavioral, and chrononutrition characteristics in adults undergoing screening for the RIO Study.

**Methods and results:**

In this cross-sectional study, 112 adults who completed the screening assessment for the RIO Study underwent anthropometric/body-composition assessment, fasting biochemistry, self-reported physical activity and sitting time, and chrononutrition evaluation. Serum adiponectin multimers were quantified by Western immunoblotting; under the present experimental conditions, only low-molecular-weight (LMW) and medium-molecular-weight (MMW) multimers were consistently detectable. Associations were assessed using Pearson correlations, adjusted multiple linear regression, and principal component analysis (PCA). Exploratory correlations suggested associations of MMW adiponectin and the MMW/LMW ratio with several anthropometric and metabolic variables. However, after multivariable adjustment, most associations with adiposity and lipid variables were attenuated, whereas inverse associations with GGT and ALT persisted. MMW adiponectin remained inversely associated with GGT (β=−0.37, p < 0.001) and ALT (β=−0.32, p = 0.002). Physically active participants had higher MMW adiponectin and MMW/LMW ratios than inactive participants (FDR-adjusted p < 0.05). Exploratory PCA identified five components with eigenvalues >1, together explaining 66.6% of the total variance. LMW and MMW adiponectin contributed to the multivariate structure, including a shared adiponectin-related component.

**Conclusion:**

In this cardiometabolic screening cohort, MMW adiponectin and the MMW/LMW ratio showed persistent inverse associations with selected liver-enzyme measures, particularly GGT and ALT, after adjustment for potential confounders. These exploratory findings support further investigation of LMW and MMW adiponectin as metabolic biomarkers using longitudinal and interventional designs.

## Introduction

Modern lifestyles characterized by high levels of physical inactivity and prolonged sedentary behavior increase adiposity and cardiometabolic risk [1], intensifying the need for biomarkers to assess metabolic health accurately [2]. In this context, circulating adiponectin has emerged as a promising biomarker of metabolic health across diverse populations. In obesity-related disorders, adiponectin has been consistently highlighted as a useful biomarker [3]. Recent studies have associated circulating adiponectin levels with various clinical conditions, including renal dysfunction [4], preeclampsia [5], Alzheimer’s disease [6], and insulin resistance [7]. However, much of the existing literature, particularly cross-sectional research, continues to rely on total adiponectin rather than multimer-specific measurements. This limitation may contribute to inconsistent findings. For example, in a cohort of 274 adults from Korea, adiponectin levels were lower in individuals with multiple metabolic syndrome components, yet multimers were not assessed [8]. Conversely, another study in a similar population reported no differences in total adiponectin between individuals with metabolic syndrome and healthy controls [9], highlighting the lack of specificity inherent in using total adiponectin alone.

Adiponectin is a ~ 30 kDa protein that forms multimers of different molecular weights, low-molecular-weight (LMW, trimer), medium-molecular-weight (MMW, hexamer), and high-molecular-weight (HMW, multimer) complexes [10]. Functionally, adiponectin acts as an insulin sensitizer, promotes fatty acid oxidation, and exerts anti-inflammatory effects [11]. Higher-molecular-weight adiponectin forms, particularly HMW and MMW multimers, are generally considered to display greater biological activity [12]. Accordingly, current recommendations emphasize measuring adiponectin multimers rather than total adiponectin concentration alone [13], as each multimer may show distinct biological activity and associations with metabolic outcomes [14]. This distinction is particularly important given previous work from our group showing that lifestyle-related interventions such as high-fat diet feeding induce marked decreases in adiponectin levels in several tissues, including skeletal muscle [15] and heart [16], that are specific to the HMW multimer.

Because adiponectin bioactivity depends not only on total abundance but also on multimer assembly, the relative distribution of LMW and MMW multimers may provide information on adipocyte secretory function, post-translational processing, and systemic lipid handling. In this context, the MMW/LMW ratio may be interpreted as a proxy of multimeric redistribution rather than as a simple concentration marker.

Given these gaps, the present study aimed to determine whether circulating LMW and MMW adiponectin and their relative pattern are associated with lipid, liver-enzyme, anthropometric, behavioral, and chrononutrition characteristics among adults undergoing cardiometabolic screening for the RIO Study.

## Materials and methods

### Study design

This is a cross-sectional study, derived from the screening process of the RIO-Study (Response to an Intervention with Omega-3 Fatty Acids on Lipid and Inflammatory Profiles in Overweight and Obese Individuals with Hypertriglyceridemia in Valdivia, Chile) [17]; registered in ClinicalTrials.gov, ID: NCT06538324; registration date: 2024-08-01. Before enrolment, all individuals provided written informed consent. Recruitment took place between July 2024 and June 2025 at Universidad San Sebastián in Valdivia, Chile. Ethical approval was granted by the Scientific Ethics Committee of Universidad San Sebastián (CEC-USS, No. 42−24, 11 April 2024), and subsequent protocol amendments were overseen by the Scientific Ethics Committee of the Los Ríos Health Service (No. 186, 12 June 2024).

Participants were recruited through social media, posters, local radio announcements, and printed graphics displayed throughout the city. Informative sessions were held to explain the study objectives, procedures, potential risks, and benefits, and to address participants’ questions before written informed consent was obtained. After consenting, participants attended a screening visit to verify eligibility according to the predefined inclusion and exclusion criteria. As part of the consent process, participants also signed an agreement authorizing the storage of their biological samples (serum) at Universidad San Sebastián (Valdivia, Chile) for future analyses.

### Participants

The present cross-sectional analysis included 112 adults who completed the screening visit for the RIO Study and had the measurements required for the current analyses. Inclusion in this cross-sectional analysis did not require participants to subsequently fulfill all eligibility criteria for the intervention phase of the parent RIO Study. Among the 112 participants included in the present analysis, BMI ranged from 21.9 to 46.1 kg/m² and fasting triglycerides from 38 to 347 mg/dL. For progression to the randomized intervention phase of the RIO Study, participants were required to be 18–65 years old, have a BMI of 25.0–34.9 kg/m² and fasting triglycerides of 100–215 mg/dL, and fulfill the remaining predefined eligibility criteria. Of the 112 participants included in the present cross-sectional analysis, 40 ultimately fulfilled the trial eligibility criteria and entered the intervention phase. Exclusion criteria included the following: BMI < 25 or >34.9 kg/m²; significant weight change (≥10% in the previous three months); fasting triglycerides >215 mg/dL; blood pressure >140/90 mmHg; presence of uncontrolled metabolic or chronic diseases (hypertension, dyslipidemia, type 2 diabetes); pregnancy or lactation; allergies or intolerances to study-related products (lactose, wheat, seaweed, or sunflower oil); use of triglyceride-lowering medications (e.g., fibrates) or anticoagulants; recent blood donation (within the past three months); and excessive alcohol consumption (≥40 g/day) (Fig 1).

**Fig 1:**
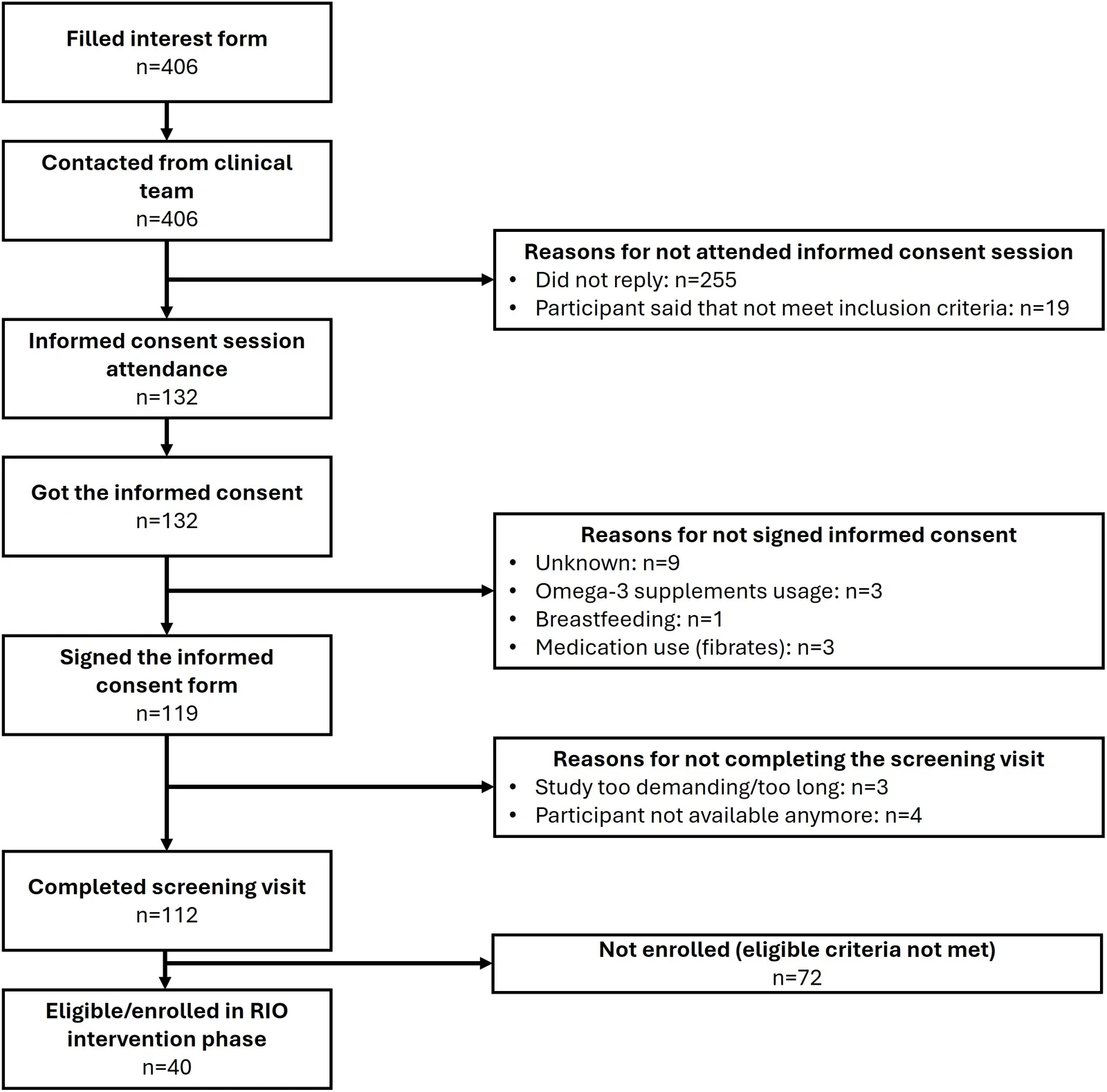
Participant flow diagram for the screening cohort and subsequent RIO Study eligibility assessment.

### Procedures

Blood pressure was assessed after a 5-min seated rest using a calibrated automated sphygmomanometer (Bokang BK6022) on the non-dominant arm, after which resting heart rate was also registered. Three readings were obtained at 2-min intervals, and the mean of the second and third measurements was recorded. For blood sample collection, the antecubital area was disinfected with 70% alcohol for at least 30 seconds before venipuncture, and serum separator tubes (BD Vacutainer® SST™, no. 367983) were filled. Tubes were centrifuged 30 minutes after collection (1500 g for 15 min), and aliquots (0.5 mL) were stored at −80 °C until analysis.

Body composition (lean mass, fat percentage, and basal metabolic rate) and body weight were measured using a bioelectrical impedance analyzer (Rice Lake X-Contact 350) while participants were barefoot and wearing light clothing; 0.8 kg was subtracted to account for clothing weight. Height was measured to the nearest 1 mm using a stadiometer (SECA 213), with participants standing in the Frankfurt plane without shoes or headgear.

As part of the Case Report Form [18], participants completed questionnaires assessing mealtime habits (chrononutrition variables: eating midpoint: clock time halfway between a person’s first and last eating event of the day: first eating time + [(last eating time − first eating time)/2] and eating jet lag: shift in meal timing between workdays/weekdays and free days/weekends, typically defined as the difference between the eating midpoint on weekends (or non-workdays) and on weekdays (or workdays)), and physical activity levels in metabolic equivalent of task (MET)*minutes/week via the International Physical Activity Questionnaire (IPAQ) as well as the daily sitting time [19].

### Laboratory tests

Serum biochemical parameters were measured using the Mindray BS-200 Chemistry Analyzer (Valtek), which operates with a photometric detection system covering wavelengths from 340 to 700 nm. This system quantifies absorbance generated by the reaction between the sample and the corresponding reagents (Shenzhen Mindray Biomedical Electronics Co., Ltd., 2006). The following analytical methods and reagents were used: glucose (GOD-PAP, ref. 200150, Valtek), creatinine (enzymatic, ref. 200095, Valtek), urea (Urease UV, ref. 200230, Valtek), uric acid (UOD-PAP, ref. 200010, Valtek), total and direct bilirubin (Acid Diazo, ref. TBI0102/DBI0102, Valtek), total protein (Biuret, ref. 200180, Valtek), albumin (BCG, ref. 200020, Valtek), alkaline phosphatase (IFCC kinetic, ref. 200120, Valtek), Gamma-Glutamyl Transferase (GGT) (Szasz method, ref. 200140, Valtek), Aspartate Aminotransferase (AST) (IFCC, ref. 105-000815-00, Mindray), and Alanine Transaminase (ALT) (IFCC, ref. 200250, Valtek).

Additionally, samples were analyzed at the Clinical Laboratory of Universidad Austral de Chile, where lipid profiles (Colestat and TG Color, Wiener Lab®) were quantified.

### Adiponectin multimers and western immunoblotting

Circulating adiponectin multimers were quantified using Western immunoblotting. For each sample, 0.5 µL of serum (under non-reducing and non-denaturing conditions) was mixed with 2.5 µL of 6X loading buffer and 12 µL of RIPA buffer (Abcam®, catalog no. ab156034), then loaded onto an acrylamide gradient gel 4–15% (Bio-Rad®, catalog no. 4561086). Following electrophoresis (80 V for the first 10 minutes and 120 V for the rest of the run, aproximately 60 minutes), proteins were transferred onto a nitrocellulose membrane (Bio-Rad®, catalog no. 1704158) using a semi-dry transfer system (Trans-Blot® Turbo™, Bio-Rad®, USA). Membranes were blocked for one hour with 5% bovine serum albumin diluted in TBST buffer. After blocking, membranes were washed in TBST and incubated overnight at 4°C with a primary antibody against human adiponectin (1:1000 dilution; rabbit monoclonal antibody, Cell Signaling®, catalog no. 2789S). The next day, membranes were washed and incubated for one hour at room temperature with a peroxidase-conjugated secondary antibody (1:2500 dilution; anti-rabbit IgG, Cell Signaling®, catalog no. 7074S). Signal detection was performed using a chemiluminescent substrate (Clarity™ Western ECL, Bio-Rad®, catalog no. 170–5061) and visualized with a densitometric imaging system (G:Box, Syngene®, USA). Band intensities were quantified using ImageJ software. Total protein loading was assessed by Ponceau Red staining of the entire membrane. Under these immunoblotting conditions, only LMW (~90–100 kDa) and MMW (~150–160 kDa) multimers were consistently detected, whereas the HMW multimer remained below the detection limit (see S1 File). It is worth mentioning that this method has successfully detected HMW multimer in plasma samples from a younger cohort [20]. To ensure the quality of our western immunoblotting and decrease its inter-assay variability we: 1) processed serum samples under non-denaturing conditions, 2) loaded at equal serum volume per lane, 3) each gel included a molecular weight marker to facilitate alignment across runs, 4) membrane transfer efficiency was verified for every blot using total protein staining (Ponceau S), 5) densitometry was performed using identical background subtraction and exposure settings constrained to the linear range (i.e., avoiding saturated bands), 6) multimer signals were normalized to total lane protein and expressed in arbitrary units, and 7) all analyses were conducted blinded to participant characteristics.

### Statistical analysis

Descriptive statistics are reported as mean ± standard deviation. Differences between men and women were examined using independent-samples t tests. Pearson correlation coefficients were calculated to assess associations of adiponectin multimers (LMW, MMW) and their ratio (MMW/LMW) with anthropometric, biochemical, physical activity, and chrononutrition variables; these correlations were considered exploratory. To examine whether the principal continuous associations were independent of potential confounding, multivariable linear regression analyses were performed with MMW adiponectin and the MMW/LMW ratio as dependent variables. Both outcomes were natural-log transformed because of right-skewed distributions. Triglycerides, VLDL-C, AST, ALT, and GGT were also log-transformed where appropriate. Continuous predictors were standardized, and standardized regression coefficients (β) with 95% confidence intervals were reported. Models examining anthropometric and body-composition variables were adjusted for age, sex, and smoking status, whereas models examining biochemical and metabolic variables were additionally adjusted for BMI. BMI was not included when BMI itself or closely related adiposity variables were the predictor of interest to avoid overadjustment and collinearity. Heteroskedasticity-consistent HC3 robust standard errors were used. Sex-stratified sensitivity analyses and predictor-by-sex interaction terms were used to examine potential effect modification by sex. Group comparisons of adiponectin multimers across behavioral and metabolic phenotypes, physical activity (active vs. inactive: < 600 MET·min·week^-1^), sedentary behavior (not prolonged vs. prolonged: ≥ 8 h/day), HDL-C status (normal vs. low: < 40 mg/dL), triglycerides (normal vs. high: ≥ 150 mg/dL), and fasting glycemia (normal vs. high: ≥ 100 mg/dL), were evaluated using multiple linear regression models adjusted for age, sex, BMI, and smoking status. Principal component analysis (PCA) was performed as an exploratory multivariate analysis to examine patterns of covariation among adiponectin and metabolic characteristics. To minimize mathematical and biological redundancy, LMW and MMW adiponectin were included, whereas the derived MMW/LMW ratio was excluded. Waist circumference was selected to represent adiposity, and ApoB, triglycerides, and HDL-C were retained as complementary lipid measures; total cholesterol, LDL-C, VLDL-C, and non-HDL-C were excluded because of their close mathematical or biological relationships with the retained variables. Glucose, AST, ALT, GGT, uric acid, eating midpoint, and eating jetlag were also included. PCA was performed using the correlation matrix with listwise exclusion of missing observations; thus, variables were standardized with respect to their variance. No rotation was applied. Components with eigenvalues >1 were retained, and loadings with an absolute value ≥0.40 were considered meaningful. Components were interpreted as exploratory patterns rather than validated latent constructs. Given the exploratory nature and number of comparisons, the Benjamini–Hochberg procedure was used to control the false discovery rate within each predefined analysis family. FDR-adjusted q values <0.05 were considered statistically significant, while nominal p values are also reported for transparency. Statistical analyses were conducted using SPSS version 22 (IBM Corp., Armonk, NY, USA) and GraphPad Prism version 8 (GraphPad Software, San Diego, CA, USA).

## Results

A total of 112 participants were analyzed (mean age 40.6 ± 11.6 years). Men showed higher height, weight, lean mass, waist circumference, systolic blood pressure, vigorous physical activity, and sedentary time (all p < 0.01), whereas women had higher fat mass percentage and resting heart rate (p < 0.01). BMI, eating midpoint, and eating jetlag did not differ by sex (Table 1).

**Table 1:** Phenotypic characteristics of the participants.

Parameter	All (n = 112)	Men (n = 43)	Women (n = 69)	p-value (by sex)
Age (years)	40.6 (11.6)	38.2 (11.8)	42.1 (11.2)	0.085
Height (cm)	164 (8.9)	172 (6.9)	158 (5.2)	<0.001
Weight (kg)	83 (15.8)	91 (14.7)	77 (14.0)	<0.001
BMI (kg/m^2^)	30.8 (5.01)	31.0 (4.37)	30.7 (5.40)	0.712
Lean mass (kg)	52.9 (10.8)	63.0 (8.99)	46.6 (5.84)	<0.001
Fat mass (%)	36.4 (6.47)	31.1 (5.30)	39.7 (4.75)	<0.001
Waist circumference (cm)	95.0 (12.2)	100 (10.8)	91.6 (11.9)	<0.001
Hip circumference (cm)	107 (8.75)	107 (7.34)	108 (9.55)	0.624
Waist-to-hip ratio	0.94 (0.08)	0.99 (0.08)	0.92 (0.07)	<0.001
SBP (mmHg)	125 (13.5)	130 (12.0)	122 (13.4)	0.001
DBP (mmHg)	77.4 (9.78)	79.5 (9.93)	76.2 (9.53)	0.085
Heart rate (beats/min)	69.6 (9.15)	66.3 (8.43)	71.6 (9.05)	0.002
Vigorous PA (METs*min*wk)	686 (1296)	1296 (1731)	301 (702)	0.001
Moderate PA (METs*min*wk)	626 (788)	503 (611)	704 (877)	0.194
Walking PA (METs*min*wk)	434 (527)	464 (596)	416 (484)	0.636
Sitting time (daily hours)	7.31 (2.35)	8.10 (2.28)	6.83 (2.27)	0.006
Eating midpoint (hours)	14.8 (1.3)	14.8 (1.5)	14.8 (1.1)	0.999
Eating jetlag (hours)	0.88 (0.82)	0.83 (0.72)	0.90 (0.88)	0.666

Data is expressed as mean (SD).

Abbreviation list: BMI: body mass index; SBP: systolic blood pressure; DBP: diastolic blood pressure; PA: physical activity; MET: metabolic equivalent of task.

Metabolically, men had higher triglycerides, VLDL-C, AST, GGT, urea, creatinine, albumin, total/direct bilirubin, and uric acid, while women had higher HDL-C, MMW adiponectin, and MMW/LMW ratios (all p < 0.05). LMW adiponectin did not differ between sexes (Table 2).

**Table 2:** Circulating metabolic parameters.

Parameter	All (n = 112)	Men (n = 43)	Women (n = 69)	p-value (by sex)
ApoB (mg/dl)	81.7 (21.9)	85.0 (23.7)	79.7 (20.6)	0.233
Triglycerides (mg/dl)	140 (76.2)	168 (84.7)	122 (64.9)	0.004
Total cholesterol (mg/dl)	197 (39.4)	194 (39.7)	199 (39.4)	0.577
HDL-C (mg/dl)	52.8 (12.8)	46.9 (9.39)	56.6 (13.3)	<0.001
LDL-C (mg/dl)	119 (35.5)	119 (40.4)	118 (32.4)	0.876
VLDL-C (mg/dl)	28.0 (15.3)	33.7 (17.2)	24.4 (13.0)	0.004
Non-HDL-C (mg/dl)	144 (38.3)	147 (41.0)	142 (36.8)	0.543
Glucose (mg/dl)	93 (12.8)	95 (10.8)	91 (13.6)	0.130
Urea (mg/dl)	30 (8.7)	34 (9.5)	28 (7.3)	<0.001
Creatinine (mg/dl)	0.51 (0.20)	0.65 (0.21)	0.42 (0.12)	<0.001
AST (UI/L)	22 (9.9)	25 (12.1)	20 (8.0)	0.017
GGT (UI/L)	31 (21.6)	38 (26.9)	27 (16.2)	0.007
ALT (UI/L)	19 (9.9)	21 (8.2)	18 (10.7)	0.112
Alkaline phosphatase (UI/L)	72 (19.9)	76 (18.9)	70 (20.5)	0.196
Total proteins (g/dl)	7.2 (0.7)	7.3 (0.8)	7.2 (0.6)	0.559
Albumin (g/dl)	4.3 (0.3)	4.4 (0.3)	4.2 (0.3)	<0.001
Total bilirubin (mg/dl)	0.51 (0.29)	0.60 (0.38)	0.46 (0.21)	0.028
Direct bilirubin (mg/dl)	0.16 (0.06)	0.19 (0.08)	0.15 (0.05)	0.002
Uric acid (mg/dl)	5.2 (1.4)	6.1 (1.5)	4.7 (1.0)	<0.001
LMW adiponectin (band intensity/Ponceau)	0.22 (0.08)	0.20 (0.08)	0.23 (0.08)	0.093
MMW adiponectin (band intensity/Ponceau)	0.03 (0.02)	0.03 (0.02)	0.04 (0.03)	0.001
MMW/LMW adiponectin ratio	0.16 (0.10)	0.13 (0.12)	0.17 (0.09)	0.042

Data is expressed as mean (SD).

Abbreviation list: ApoB: apolipoprotein B; HDL: high-density lipoprotein; LDL: low-density lipoprotein; VLDL: very-low-density lipoprotein; AST: aspartate aminotransferase; GGT: gamma-glutamyl transferase; ALT: alanine aminotransferase; LMW: low-molecular weight; MMW: medium-molecular weight

Exploratory Pearson correlations analyses suggested inverse relationships of MMW adiponectin and the MMW/LMW ratio with several anthropometric, lipid, liver-enzyme, and uric-acid variables, although the magnitude of these associations varied substantially across individual measures (Fig 2). In contrast, LMW adiponectin showed weaker and more heterogeneous correlations with metabolic variables. Participants with low HDL-C (<40 mg/dL) or high triglycerides (≥150 mg/dL) showed lower MMW adiponectin and lower MMW/LMW ratios, whereas fasting glycemia (≥100 mg/dL) did not differentiate adiponectin multimer levels (Fig 3).

**Fig 2:**
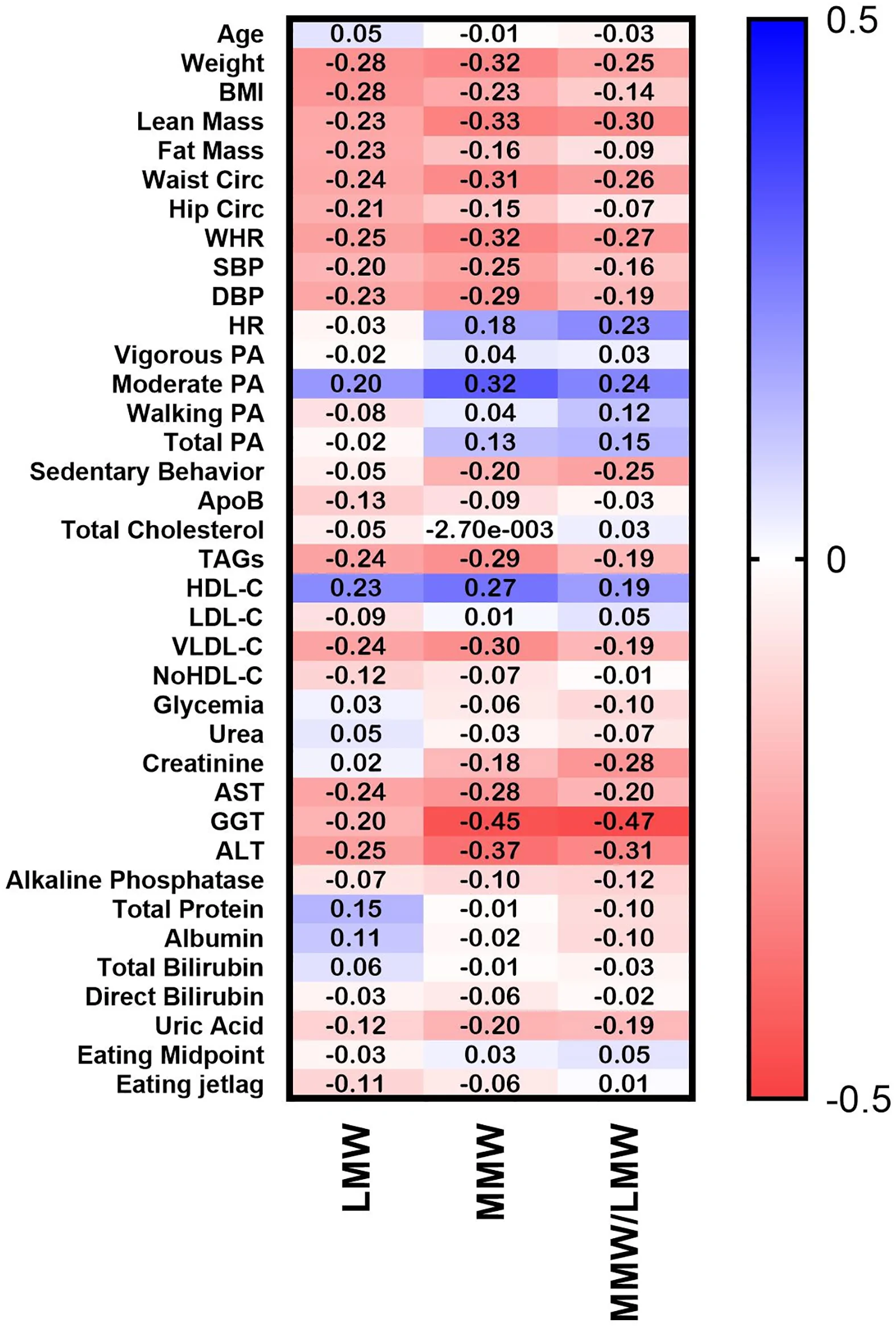
Unadjusted correlation matrix between adiponectin multimers (LMW, MMW) and their ratio (MMW/LMW) with anthropometric, hemodynamic, physical activity, lipid, hepatic, renal, and chrononutrition variables. Each cell represents the Pearson correlation coefficient (r) between a given variable (rows) and an adiponectin marker (columns). Colors indicate the direction and magnitude of the association: blue represents positive correlations and red represents negative correlations, with intensity proportional to the strength of the relationship (color scale: –0.5 to +0.5). Pearson correlations are presented as exploratory, unadjusted associations; inferential interpretation was based on the adjusted multivariable analyses reported in S1 Table.

**Fig 3:**
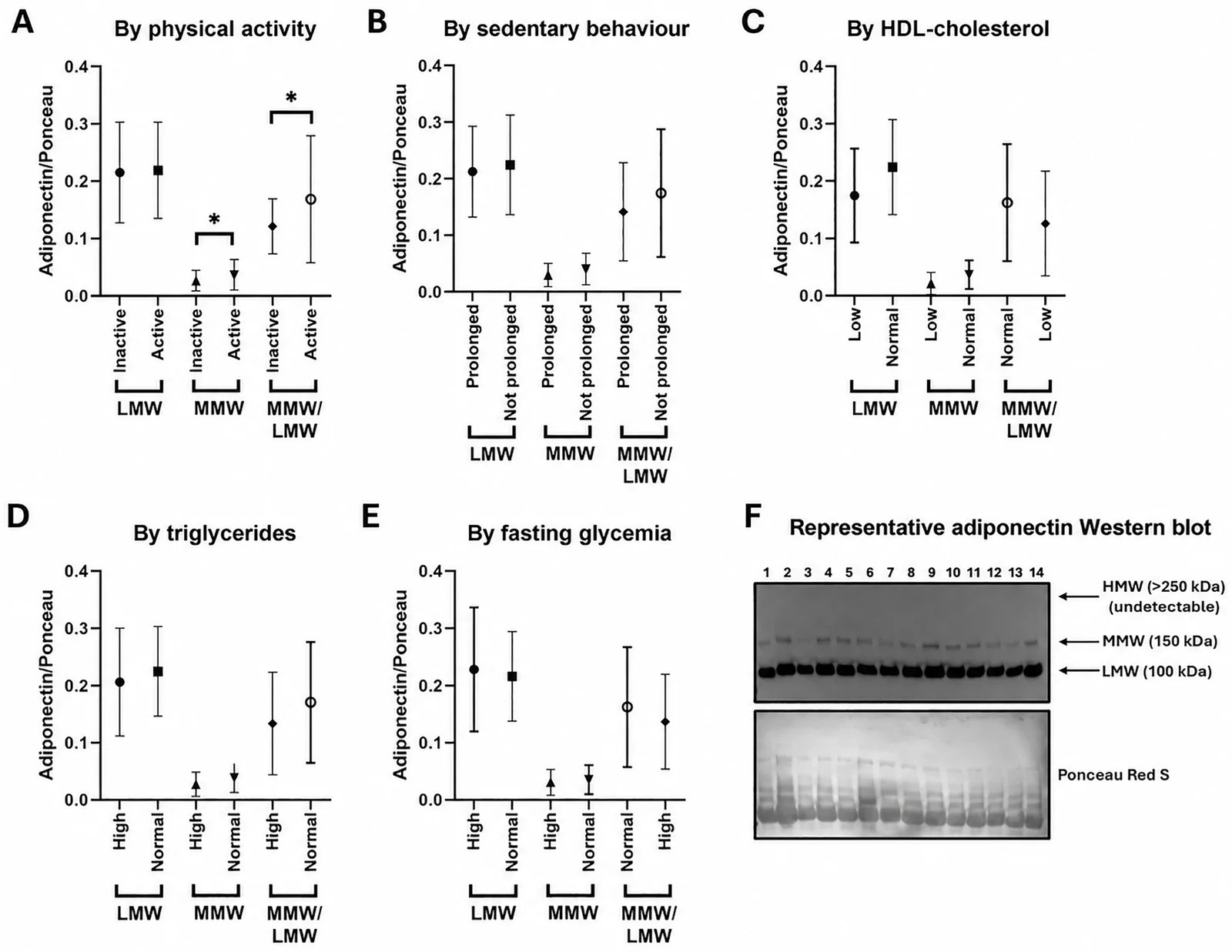
Adiponectin multimers (LMW, MMW), their ratio (MMW/LMW), and representative Western blot according to behavioral and metabolic phenotypes. (A) Physical activity: participants were classified as inactive (<600 MET·min·week^-1^) or active based on total physical activity. (B) Sedentary behavior: prolonged sedentary time was defined as ≥8 h/day of sitting. (C) HDL-cholesterol: low HDL-C was defined as <40 mg/dL. (D) Triglycerides: high triglycerides were defined as ≥150 mg/dL. (E) Fasting glycemia: high fasting glycemia was defined as ≥100 mg/dL. (F) Representative Western blot showing serum adiponectin multimers. Distinct LMW (~100 kDa) and MMW (~150 kDa) bands were consistently detected, whereas HMW (>250 kDa) remained below detection under experimental conditions. Ponceau Red S staining is included as a loading control. Each lane represents serum from a different participant; lanes are shown solely as representative examples of adiponectin multimer detection and are not arranged according to the categorical comparisons shown in panels A–E. *: indicates differences remaining statistically significant after Benjamini–Hochberg FDR correction (q < 0.05) in multiple linear regression models adjusted for age, sex, BMI, and smoking status.

In multivariable analyses, several associations observed in the unadjusted correlations were attenuated after adjustment for age, sex, smoking status, and, where appropriate, BMI (S1 Table). In particular, associations with adiposity/body-composition measures, triglycerides, VLDL-C, HDL-C, ApoB, and uric acid were no longer statistically significant. In contrast, MMW adiponectin remained inversely associated with GGT (standardized β = −0.37, 95% CI −0.58 to −0.16, p < 0.001) and ALT (β = −0.32, 95% CI −0.52 to −0.11, p = 0.002), whereas the association with AST was borderline (β = −0.20, p = 0.054). Similarly, the MMW/LMW ratio remained inversely associated with GGT (β = −0.36, p = 0.004) and ALT (β = −0.25, p = 0.009). Sex-stratified analyses showed generally stronger estimates for GGT and ALT among women; however, none of the predictor-by-sex interaction terms was statistically significant, providing no evidence of effect modification by sex (S1 Table).

When behavioral phenotypes were examined, physically active participants displayed higher MMW adiponectin levels and higher MMW/LMW ratios than inactive individuals. In contrast, prolonged sedentary behavior and high fasting glycemia were not associated with significant differences in adiponectin multimer levels (Fig 3). Eating midpoint and eating jetlag exhibited minimal correlations with adiponectin measures.

The PCA included 104 participants with complete data for the 13 non-redundant variables. Sampling adequacy was acceptable (KMO = 0.694), and Bartlett’s test of sphericity was significant (χ²[78] = 342.66, p < 0.001). Five components had eigenvalues >1 and together explained 66.6% of the total variance. PC1 was characterized by positive loadings for ALT, triglycerides, waist circumference, GGT, uric acid, AST, and ApoB and negative loadings for HDL-C, MMW adiponectin, and LMW adiponectin. PC2 was characterized primarily by positive loadings for MMW and LMW adiponectin and a negative loading for eating jetlag. PC3 included HDL-C, ApoB, AST, and waist circumference; PC4 included eating jetlag, glucose, and eating midpoint; and PC5 was characterized predominantly by eating midpoint, with ApoB loading in the opposite direction (Fig 4; Table 3). These components are interpreted as exploratory patterns of covariation rather than discrete biological constructs.

**Table 3:** Description and exploratory interpretation of the principal components (PC).

Component	Variables with meaningful loadings	Exploratory Interpretation
**PC1 (27.9%)**	ALT (+0.76); TAGs (+0.68); WC (+0.63); GGT (+0.63); uric acid (+0.59); HDL-C (−0.56); AST (+0.55); MMW (−0.51); ApoB (+0.44); LMW (−0.41)	Broad cardiometabolic–liver component. Higher waist circumference, triglycerides, ApoB, liver-enzyme levels, and uric acid co-varied with lower HDL-C and lower adiponectin signals.
**PC2 (11.9%)**	MMW (+0.60); LMW (+0.57); eating jetlag (−0.44)	Adiponectin-related component. LMW and MMW adiponectin loaded together, while eating jetlag contributed in the opposite direction.
**PC3 (10.0%)**	HDL-C (+0.54); ApoB (+0.52); AST (+0.50); WC (−0.49)	Mixed lipid–anthropometric component. This component captured covariation among HDL-C, ApoB, AST, and waist circumference without a straightforward favorable–unfavorable metabolic interpretation.
**PC4 (8.5%)**	Eating jetlag (+0.57); glucose (+0.53); eating midpoint (−0.40)	Glycemia–meal-timing component. Higher glucose and greater eating jetlag loaded together, whereas eating midpoint contributed in the opposite direction.
**PC5 (8.3%)**	Eating midpoint (+0.72); ApoB (−0.44)	Meal-timing component. Eating midpoint was the dominant contributor, with ApoB loading in the opposite direction.

PCA was performed using the correlation matrix and a reduced, non-redundant set of 13 variables. Five components with eigenvalues >1 were retained. Missing data were handled using listwise exclusion (n = 104), and no rotation was applied. Loadings with an absolute value ≥0.40 were considered meaningful for exploratory interpretation. The direction of component loadings is arbitrary; therefore, positive and negative signs indicate opposite orientation within a component rather than intrinsically favorable or unfavorable effects.

Abbreviations: LMW, low-molecular-weight adiponectin; MMW, medium-molecular-weight adiponectin; WC, waist circumference; ApoB, apolipoprotein B; TAGs, triglycerides; HDL-C, high-density lipoprotein cholesterol; AST, aspartate aminotransferase; ALT, alanine aminotransferase; GGT, gamma-glutamyl transferase.

**Fig 4:**
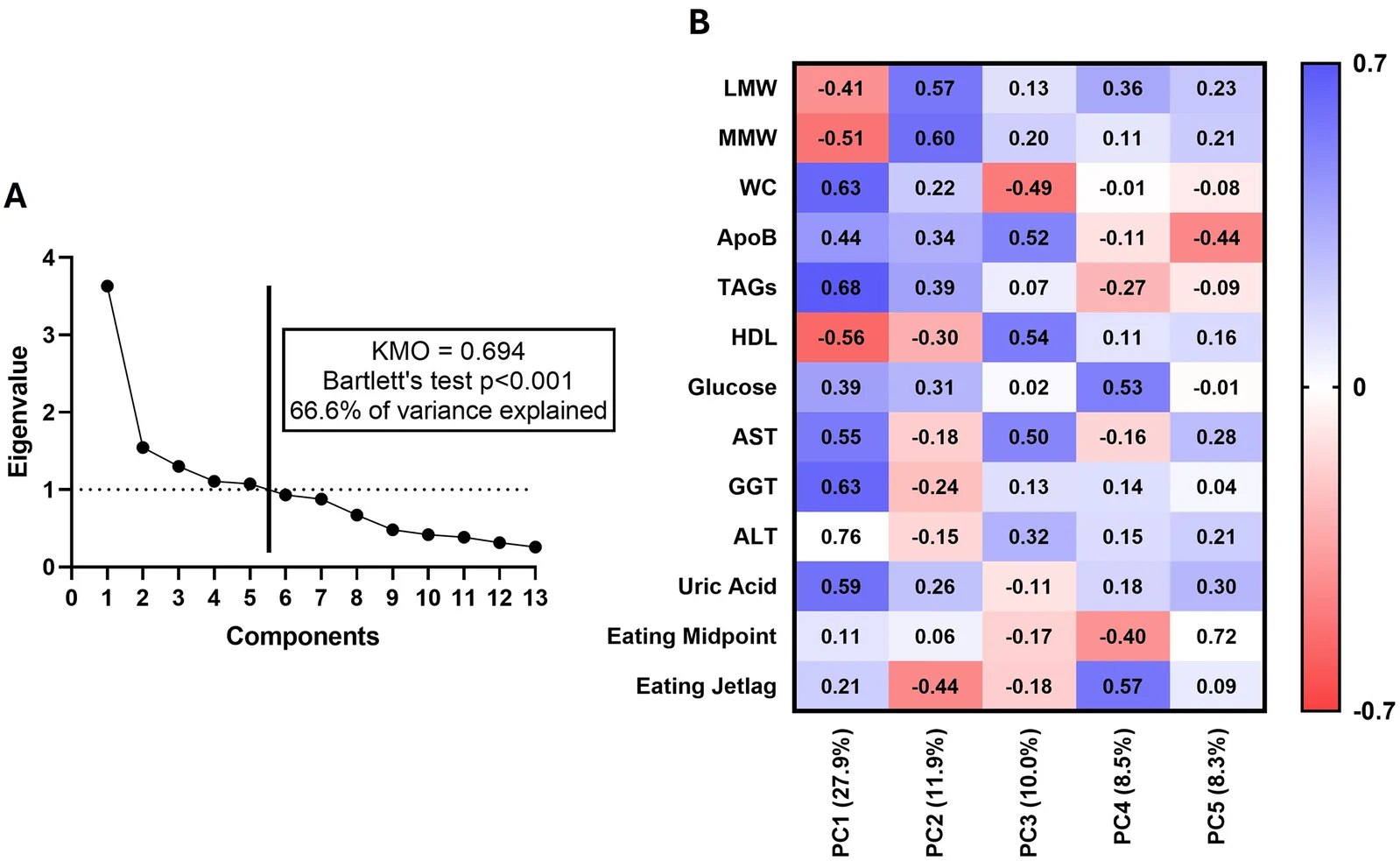
Exploratory principal component analysis of circulating adiponectin and metabolic variables. (A) Scree plot showing eigenvalues for the 13 components. Five components with eigenvalues >1 were retained, together explaining 66.6% of the total variance. Sampling adequacy was acceptable (Kaiser–Meyer–Olkin [KMO] = 0.694), and Bartlett’s test of sphericity was significant (p < 0.001). (B) Heatmap showing the unrotated component loadings for the five retained principal components. Positive loadings are shown in blue and negative loadings in red, with greater color intensity indicating larger absolute loadings. The percentage of total variance explained by each component is shown below the corresponding component. PCA was conducted using the correlation matrix and complete cases (n = 104); no rotation was applied. Abbreviations: LMW, low-molecular-weight adiponectin; MMW, medium-molecular-weight adiponectin; WC, waist circumference; ApoB, apolipoprotein B; TAGs, triglycerides; HDL, high-density lipoprotein cholesterol; AST, aspartate aminotransferase; ALT, alanine aminotransferase; GGT, gamma-glutamyl transferase.

## Discussion

The present study identified distinct unadjusted association patterns for circulating LMW and MMW adiponectin. However, multivariable analyses showed that several relationships with adiposity and lipid-related variables were attenuated after accounting for sex and other potential confounders. In contrast, the most consistent independent associations involved MMW adiponectin and the MMW/LMW ratio with GGT and ALT, whereas LMW adiponectin showed weaker and more heterogeneous associations across the metabolic phenotype. Importantly, liver-enzyme values were generally within conventional reference ranges; therefore, these inverse associations should be interpreted as reflecting variation in liver-enzyme profiles rather than evidence of liver injury or clinically overt hepatic dysfunction. In addition, physically active participants displayed higher MMW adiponectin levels and higher MMW/LMW ratios, while prolonged sedentary behavior and dyslipidemic phenotypes (low HDL-C or high triglycerides) were linked to lower MMW adiponectin. Finally, PCA provided an exploratory view of several dimensions within the metabolic phenotype. Notably, LMW and MMW adiponectin loaded strongly on a common component, whereas liver-related, adiposity–lipid, and meal-timing variables were represented predominantly on other components. This pattern indicates that variation in the measured adiponectin forms is embedded within, but not identical to, the broader metabolic phenotype. Given the exploratory nature of PCA and the component-retention approach used, these patterns should not be interpreted as evidence of discrete biological pathways and require replication in independent cohorts. Together, these findings indicate that circulating LMW and MMW adiponectin show partially differing patterns of association within the broader metabolic phenotype of this cardiometabolic screening cohort.

Historically, adiponectin has been conceptualized as a protective adipokine with antidiabetic, anti-atherosclerotic, and anti-inflammatory actions, and lower circulating levels have been consistently associated with obesity, insulin resistance, and cardiometabolic disease [21]. The recognition that adiponectin circulates as trimers (LMW), hexamers (MMW), and higher-order multimers (HMW) added a new layer of complexity, raising the question of whether all multimers share the same biological functions [22]. Early work suggested that the HMW fraction is the main driver of adiponectin’s insulin-sensitizing effects, particularly regarding hepatic and skeletal muscle glucose disposal and lipid oxidation [23]. Complementary evidence showed that a higher proportion of LMW relative to total adiponectin was associated with type 2 diabetes and insulin resistance, whereas HMW/total adiponectin (TA) ratios were reduced in individuals with metabolic dysfunction [24]. In the same study, individuals with T2D showed lower HMW/total adiponectin ratios, further supporting the concept that adiponectin multimers behave differently in the context of metabolic dysfunction. HMW adiponectin was not detectable under the present immunoblot conditions, which may reflect very low abundance in this cohort. Importantly, the observed associations for MMW adiponectin and the MMW/LMW ratio were internally consistent across complementary analyses (correlations, regression, and multivariate patterning), supporting the validity of the detected multimers; nevertheless, future studies should prioritize optimized approaches for HMW detection (e.g., validated multimer-preserving methods and/or orthogonal assays) to confirm whether inclusion of HMW further improves metabolic phenotyping.

In our study, MMW adiponectin displayed stronger and more coherent associations with cardiometabolic markers than LMW, particularly with lipid parameters and liver-related outcomes. MMW adiponectin and the MMW/LMW ratio showed partially overlapping but distinct associations with metabolic variables, with the most consistent patterns involving selected lipid and liver-enzyme measures. Importantly, not all individual associations were strong; for example, the correlations of MMW adiponectin with fat mass and ApoB were weak. These findings suggest that MMW adiponectin may be particularly informative in relation to variation in liver-enzyme profiles within this cardiometabolic screening cohort. Moreover, MMW adiponectin could be responsive to intervention, given that our group has observed that, as an acute adaptation after one session of high-intensity interval training, MMW adiponectin significantly increased in physically inactive young adults [20]. Because total adiponectin and HMW adiponectin were not quantified in the present study, direct comparison of LMW and MMW signals with total or HMW adiponectin was not possible. Future studies incorporating all major circulating adiponectin forms are required to determine their relative or incremental value for metabolic phenotyping [25]. However, although MMW adiponectin showed inverse associations with AST, ALT, and GGT, mean concentrations of these enzymes were within conventional clinical reference ranges. Accordingly, these associations should not be interpreted as evidence that MMW adiponectin identifies liver injury or clinically overt hepatic dysfunction. Rather, they indicate that variation in MMW adiponectin may accompany variation in liver-enzyme levels within this predominantly subclinical range. Given the modest magnitude of these associations and the absence of direct measures of hepatic steatosis or liver function, their biological and clinical relevance requires confirmation.

Behavioral phenotypes further highlight the sensitivity of MMW adiponectin to lifestyle exposures. Participants who self-reported as physically active showed higher MMW and MMW/LMW ratios. This aligns with evidence that regular exercise upregulates adiponectin or shifts its multimeric distribution toward more favorable forms [15,26]. Mechanistically, physical activity may promote multimerization through improved redox balance, post-translational processing, and reduced adipose inflammation [27]. It is worth mentioning that, for exploratory purposes, HDL-C status was defined using a single cutoff of 40 mg/dL for both men and women, however, future studies with larger samples should apply sex-specific HDL-C thresholds.

The revised PCA provided an exploratory view of the multivariate metabolic structure after removing mathematically and biologically redundant variables. The first component combined higher waist circumference, triglycerides, liver-enzyme levels, uric acid, and ApoB with lower HDL-C and lower LMW and MMW adiponectin signals. A second component was characterized predominantly by positive loadings of both LMW and MMW adiponectin, whereas the remaining components captured mixed lipid–anthropometric, glycemia–meal-timing, and meal-timing patterns. These findings suggest that adiponectin variation is embedded within the broader metabolic phenotype while also contributing to secondary patterns of covariation [23,28]. MMW and LMW adiponectin contributed to the broader multivariate metabolic structure. Both adiponectin forms loaded inversely on PC1, together with higher loadings for waist circumference, triglycerides, ApoB, liver enzymes, and uric acid and an opposite loading for HDL-C. In addition, MMW and LMW adiponectin loaded positively together on PC2, whereas eating jetlag loaded in the opposite direction. These findings suggest that variation in LMW and MMW adiponectin is related to, but not fully captured by, conventional cardiometabolic characteristics [29–31]. The contribution of eating jetlag to PC2 should be interpreted cautiously, given the exploratory nature of the PCA and the self-reported assessment of chrononutrition [32]. Given the exploratory PCA approach and component-retention criterion used, these components should be considered hypothesis-generating rather than evidence of discrete biological pathways.

Sex-related differences further support context-specific interpretation. Women exhibited higher HDL-C, MMW adiponectin, and MMW/LMW ratios than men, despite similar BMI, and these sex differences in adiponectin levels have been previously described [33]. Differences in fat distribution, more subcutaneous in women and more visceral in men, may also contribute [34]. Although causality cannot be inferred, these patterns suggest that adiponectin multimer interpretation may require sex-specific considerations; therefore, regression models were adjusted for sex. Sex-stratified sensitivity analyses suggested stronger inverse associations of MMW adiponectin and the MMW/LMW ratio with GGT and ALT among women. However, formal predictor-by-sex interaction tests were not significant. Thus, although sex was an important confounder of several crude associations, the present data do not provide evidence that the relationships between adiponectin multimers and the examined metabolic variables differ significantly between women and men.

As strengths of this study, we quantified LMW and MMW multimers directly using Western immunoblotting rather than total adiponectin or ELISA-based proxies, in a highly characterized population (including non-standard lifestyle variables such as chrononutrition determinations), and applied multidimensional methods such as PCA to move beyond single-variable correlations. However, this work also has limitations. First, the cross-sectional design limits the interpretation of causality behind the associations described here. Whether changes in MMW or LMW adiponectin precede or follow metabolic improvements remains unknown. Second, the inability to quantify HMW adiponectin, either due to technical limitations of Western blotting under the experimental conditions or low circulating abundance, prevents direct comparison of all three major biologically relevant multimers, an issue that should be addressed in future studies. Third, physical activity, sedentary time, and chrononutrition metrics rely on self-report, which may introduce misclassification and attenuate associations with adiponectin multimers. Fourth, no postprandial or dynamic metabolic assessments were performed, limiting the ability to capture adiponectin responsiveness to feeding or glycemic perturbations. Medication was not systematically recorded during the screening stage from which the present cross-sectional dataset was derived. Consequently, potential confounding by lipid-lowering or other metabolically relevant medications could not be examined and should be considered when interpreting the observed associations. Finally, participants were recruited from a cardiometabolic screening cohort in a single Chilean city; therefore, the findings may not generalize to other populations or ethnic and geographical settings.

## Conclusion

This study suggests that circulating LMW and MMW adiponectin exhibit distinct patterns of association with metabolic characteristics. Although several crude associations with anthropometric and lipid variables were attenuated after adjustment for potential confounders, MMW adiponectin and the MMW/LMW ratio showed persistent inverse associations with selected liver-enzyme measures, particularly GGT and ALT. These findings are exploratory and should not be interpreted as evidence of liver injury, but support further investigation of MMW adiponectin in relation to metabolic phenotype and subclinical variation in liver-enzyme profiles.

### Key points

- MMW adiponectin and the MMW/LMW ratio showed persistent inverse associations with GGT and ALT.
- Several crude adiposity and lipid associations were attenuated after adjustment for potential confounders.
- LMW and MMW adiponectin contributed to the exploratory multivariate structure, including a shared adiponectin-related component.

### Declaration of generative AI and AI-assisted technologies in the manuscript preparation process

During the preparation of this work the authors used ChatGPT to improve the English grammar. After using this tool, the authors reviewed and edited the content as needed and take full responsibility for the content of the published article.

## Supporting information

S1 File Original uncropped Western immunoblot images for circulating adiponectin multimers. Whole-membrane images corresponding to the eight gels used to analyze serum samples from the 112 participants are shown together with the corresponding Ponceau S total-protein staining. Gels 1–8 contain samples 1–14, 15–28, 29–42, 43–56, 57–70, 71–84, 85–98, and 99–112, respectively. Low-molecular-weight (LMW; ~ 100 kDa) and medium-molecular-weight (MMW; ~ 150 kDa) adiponectin multimers were consistently detected, whereas high-molecular-weight (HMW; > 250 kDa) adiponectin was not detectable under the present experimental conditions. Ponceau S staining was used to assess total protein loading and membrane transfer.
(PDF)

S1 Table Adjusted and sex-specific analyses of the associations between circulating MMW adiponectin, the MMW/LMW ratio, and metabolic variables. Multivariable regression analyses, sex-stratified sensitivity analyses, and predictor-by-sex interaction tests are presented. Standardized β coefficients, 95% confidence intervals, p values, and FDR-adjusted q values are reported.
(XLSX)
