# Supplementary material for: Circulating low- and medium-molecular-weight adiponectin are associated with metabolic phenotypes in adults undergoing cardiometabolic screening
Source: PLoS One. 2026 Oct 5;21(10):e0360041. doi: 10.1371/journal.pone.0360041 (PMC13637893; doi:10.1371/journal.pone.0360041)

Supplementary information (full blots, adiponectin isoforms)

Manuscript: **Circulating low- and medium-molecular-weight adiponectin are associated with metabolic phenotypes in adults undergoing cardiometabolic screening**

Authors: Sergio Martínez-Huenchullán, Nicole Cares, Josefina Enríquez, Consuelo Quezada, Jessica Molina, Teresa Ross, Amanda Bentes, Yanara Pavez, Paola Rubilar, Catalina Ramírez-Contreras, Monserrat Fitó, Álvaro Hernáez, Viviana Sandoval

Western immunoblotting (whole membranes)

Gel 1 (samples 1-14)

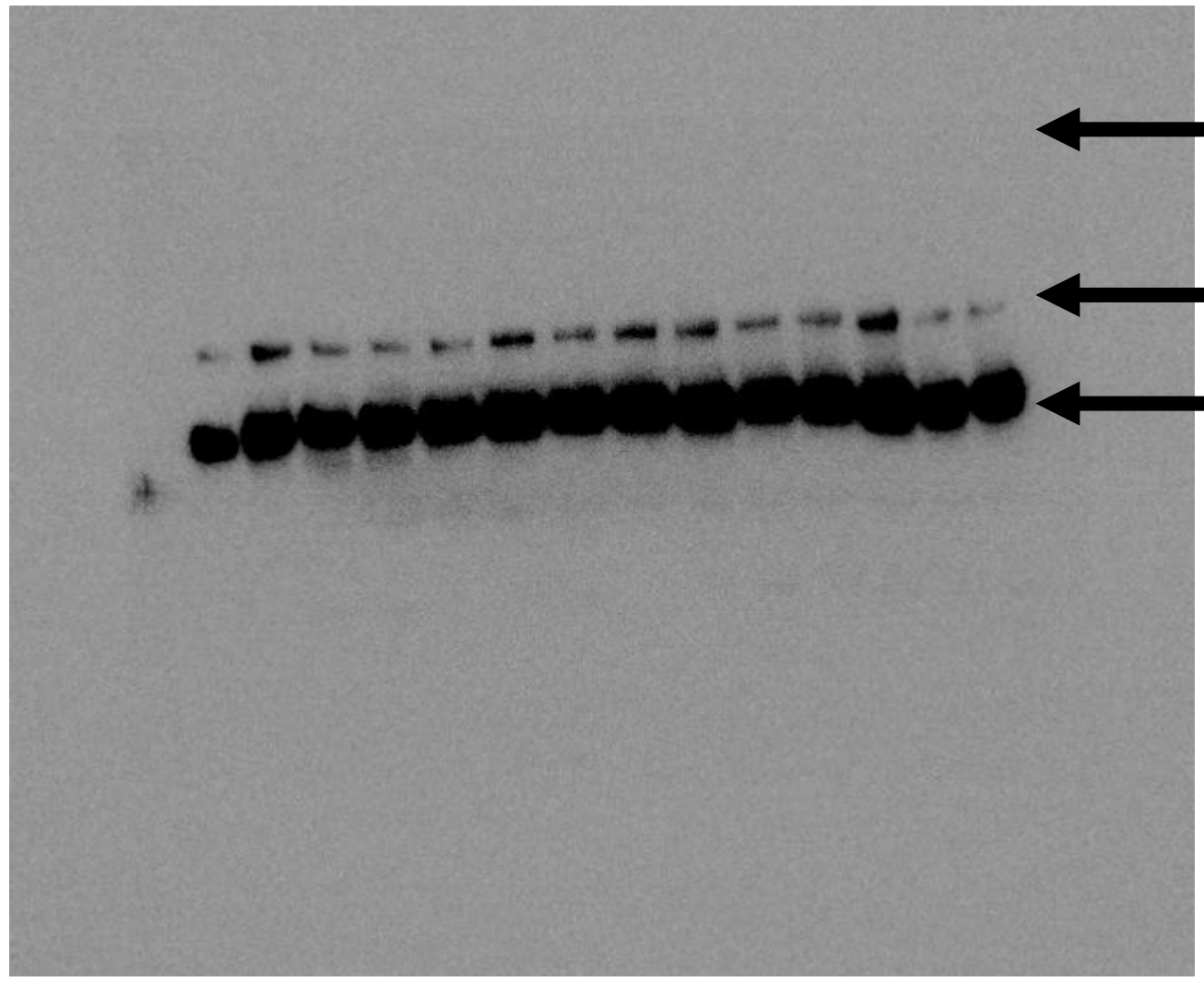

>250 kDa (HMW) (undetectable)

150 kDa (MMW)

100 kDa (LMW)

Gel 2 (samples 15-28)

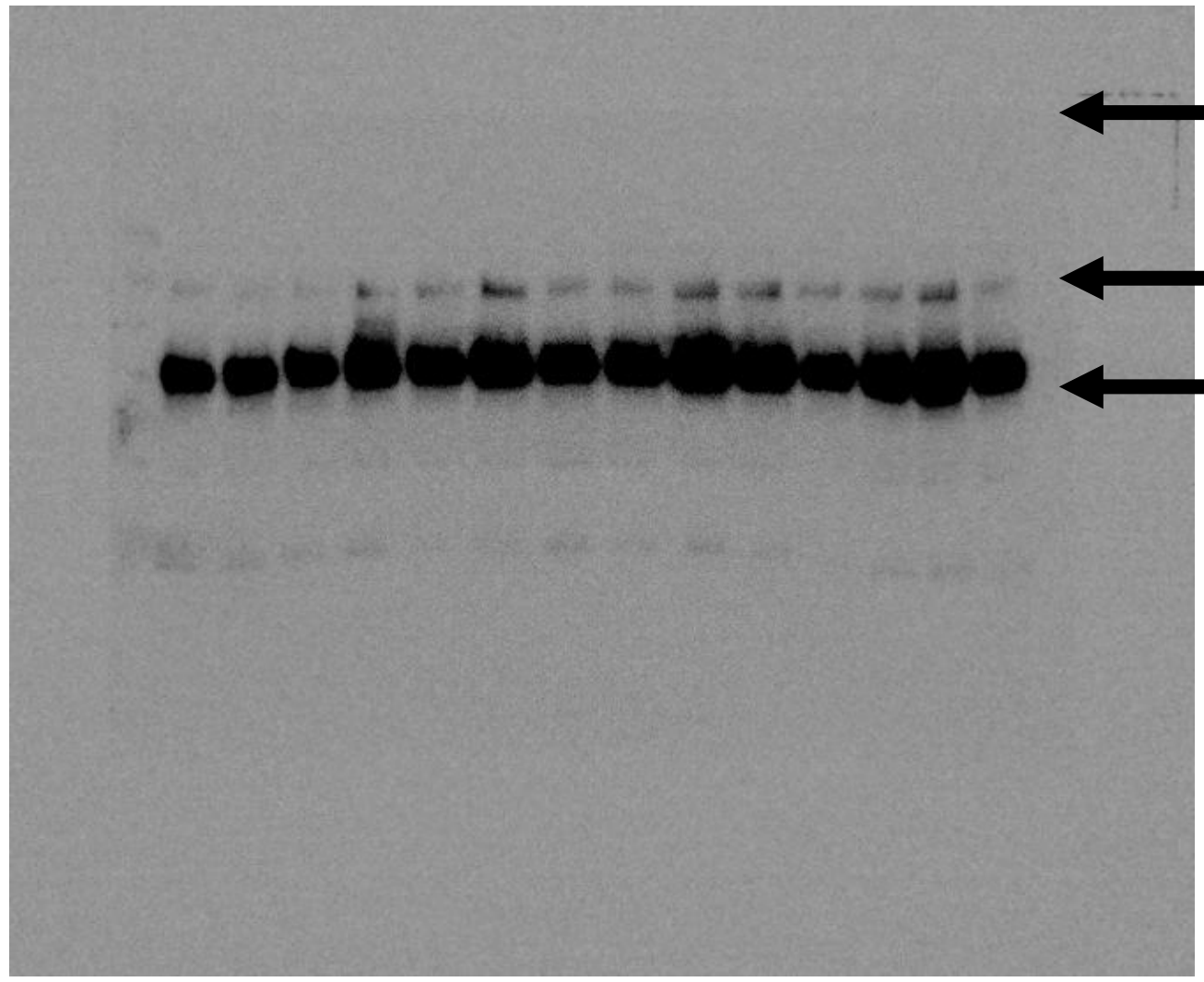

>250 kDa (HMW) (undetectable)

150 kDa (MMW)

100 kDa (LMW)

Ponceau 1

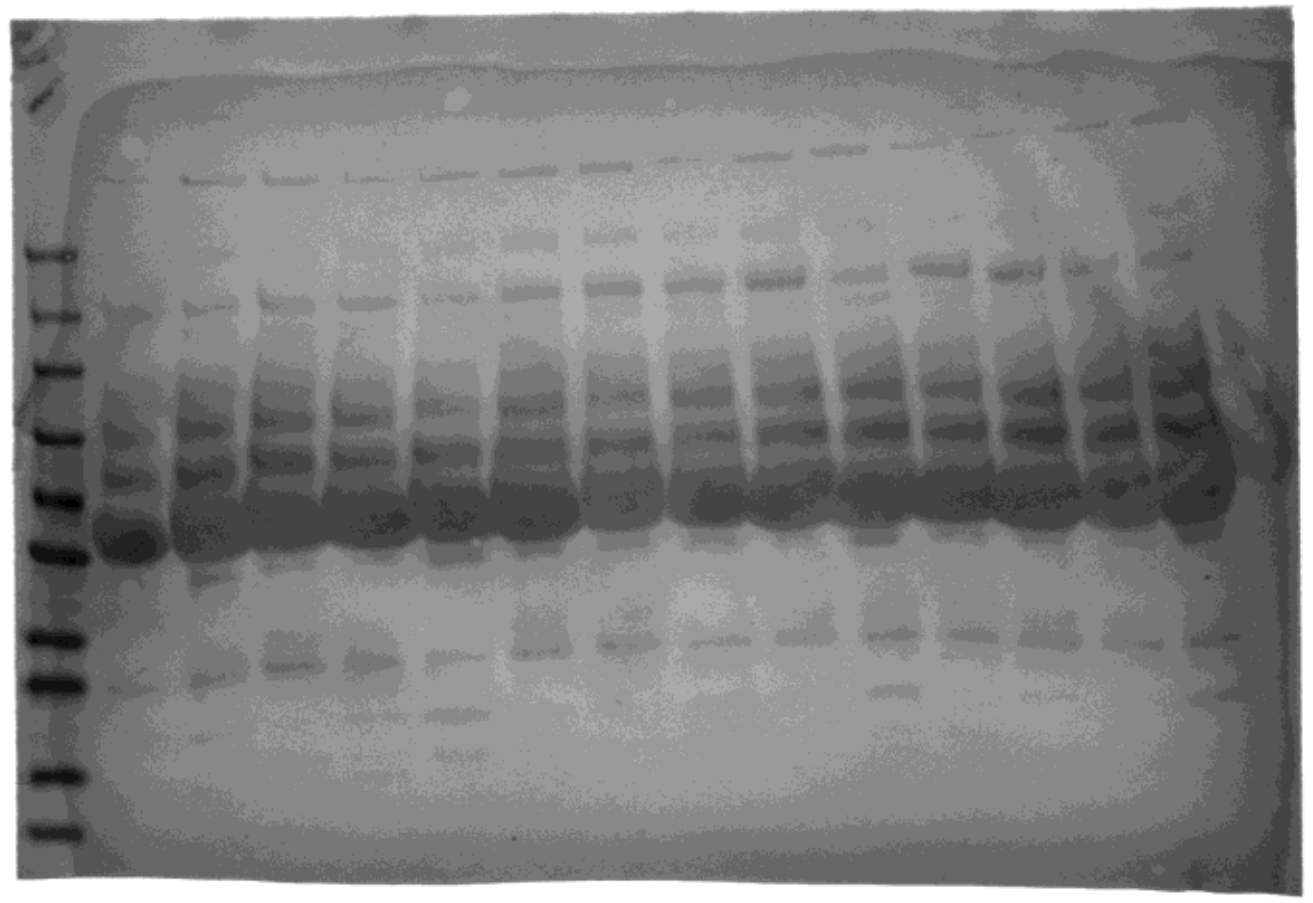

Ponceau 2

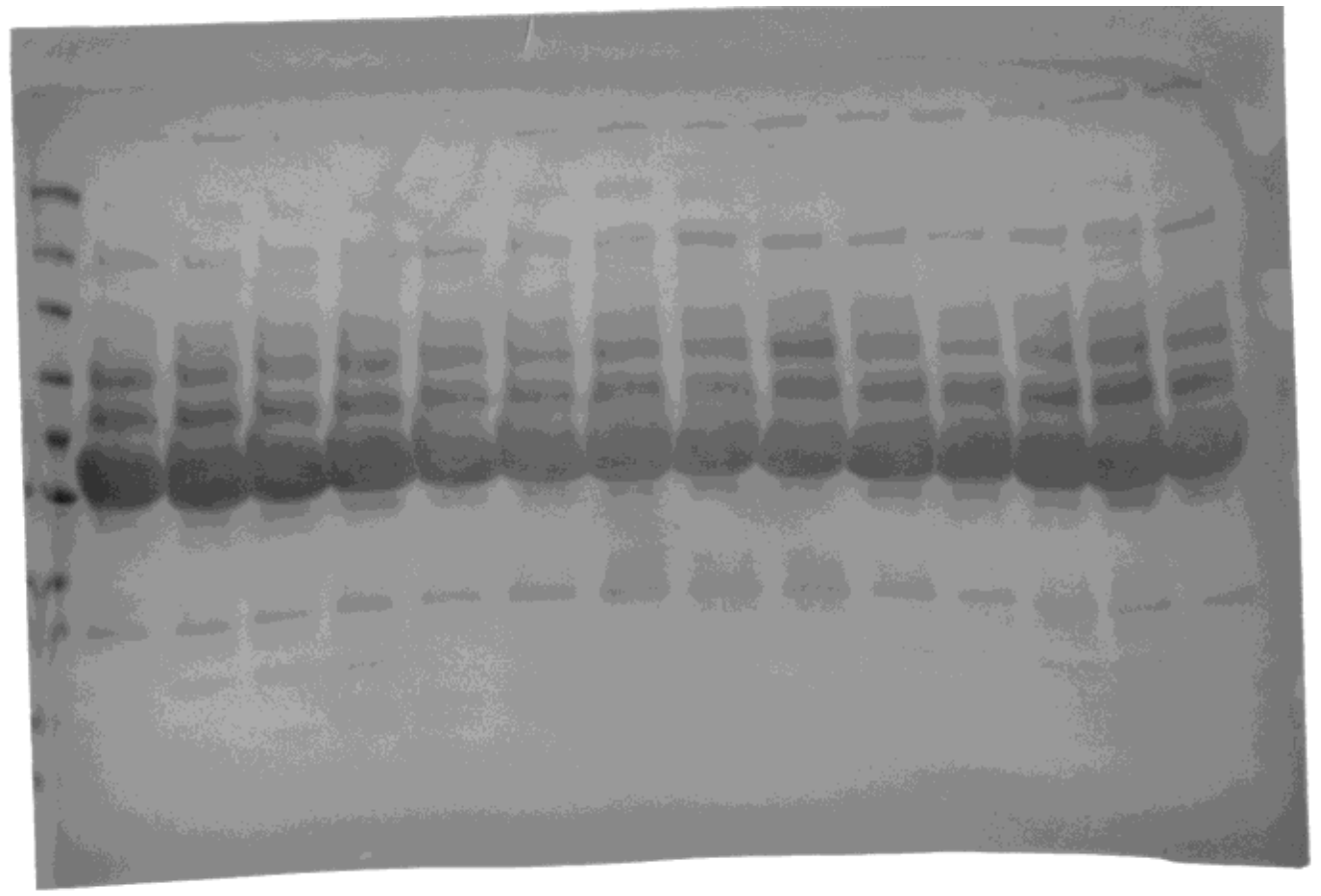

Western immunoblotting (whole membranes)

Gel 3 (samples 29-42)

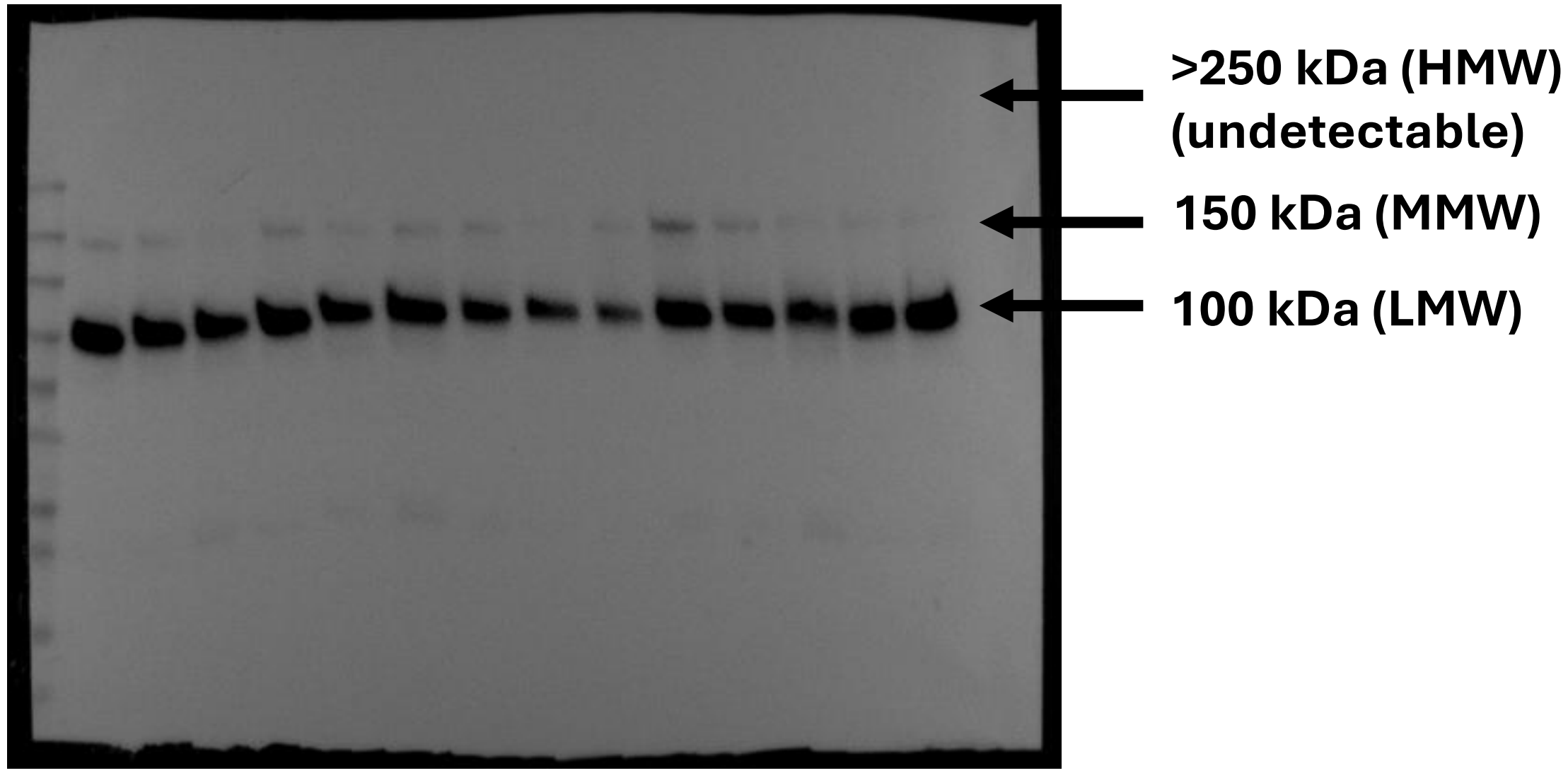

Gel 4 (samples 43-56)

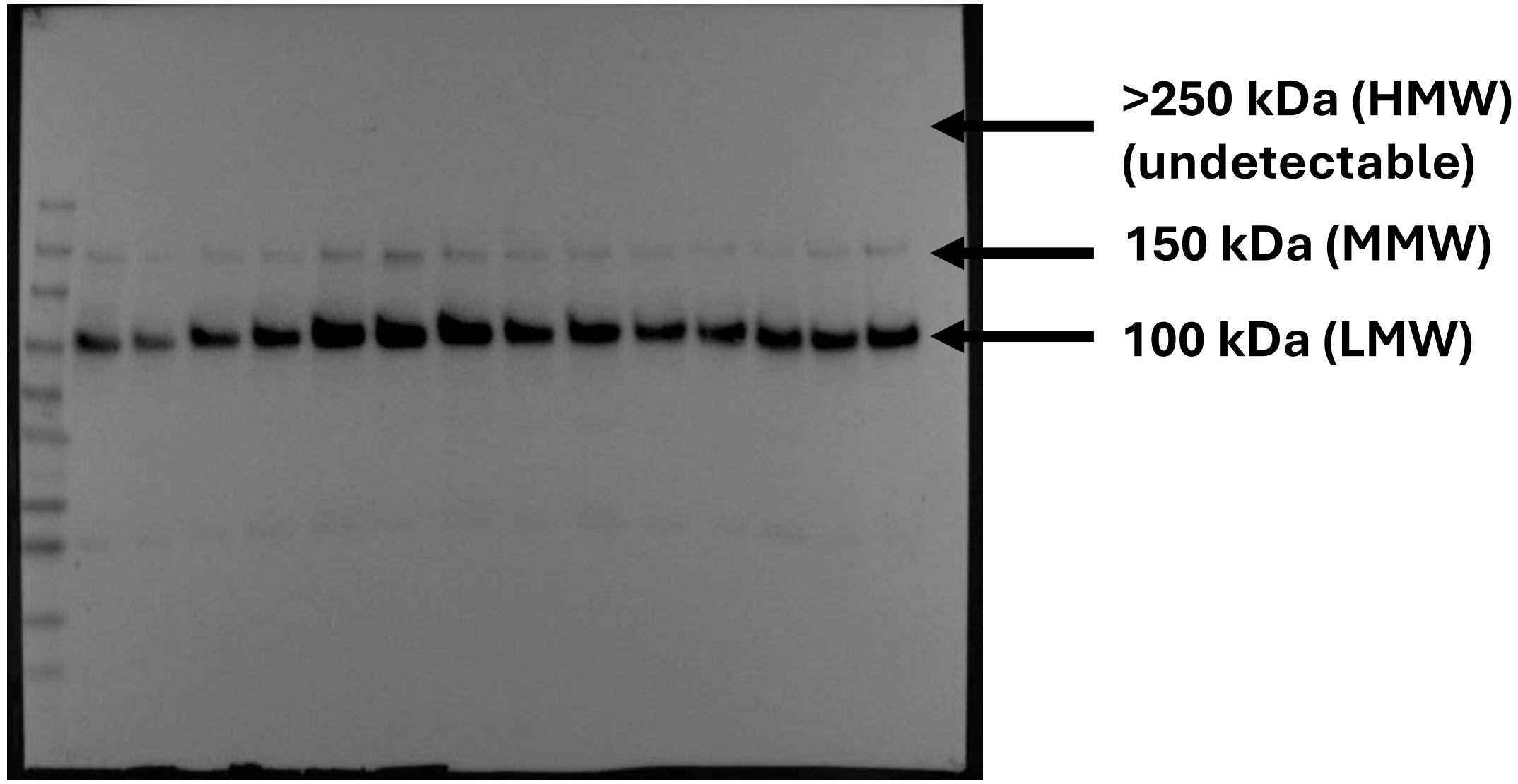

Ponceau 3

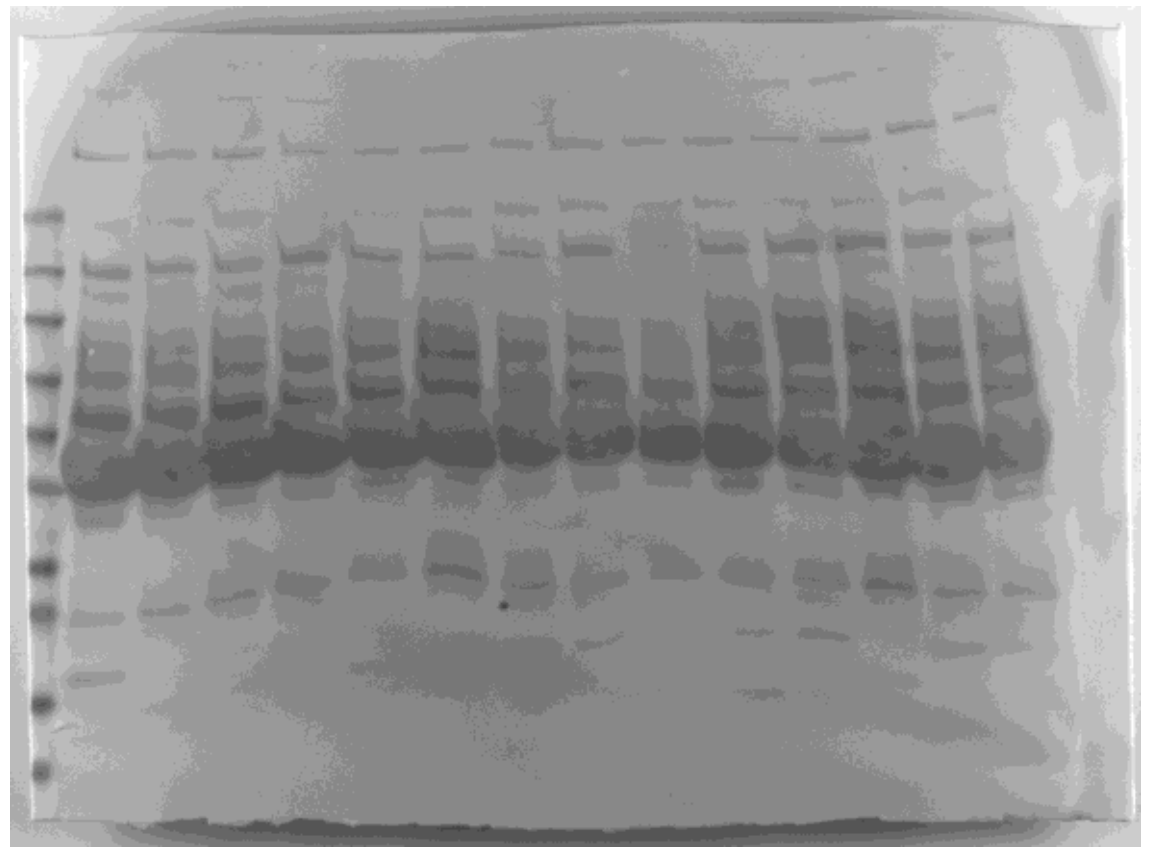

Ponceau 4

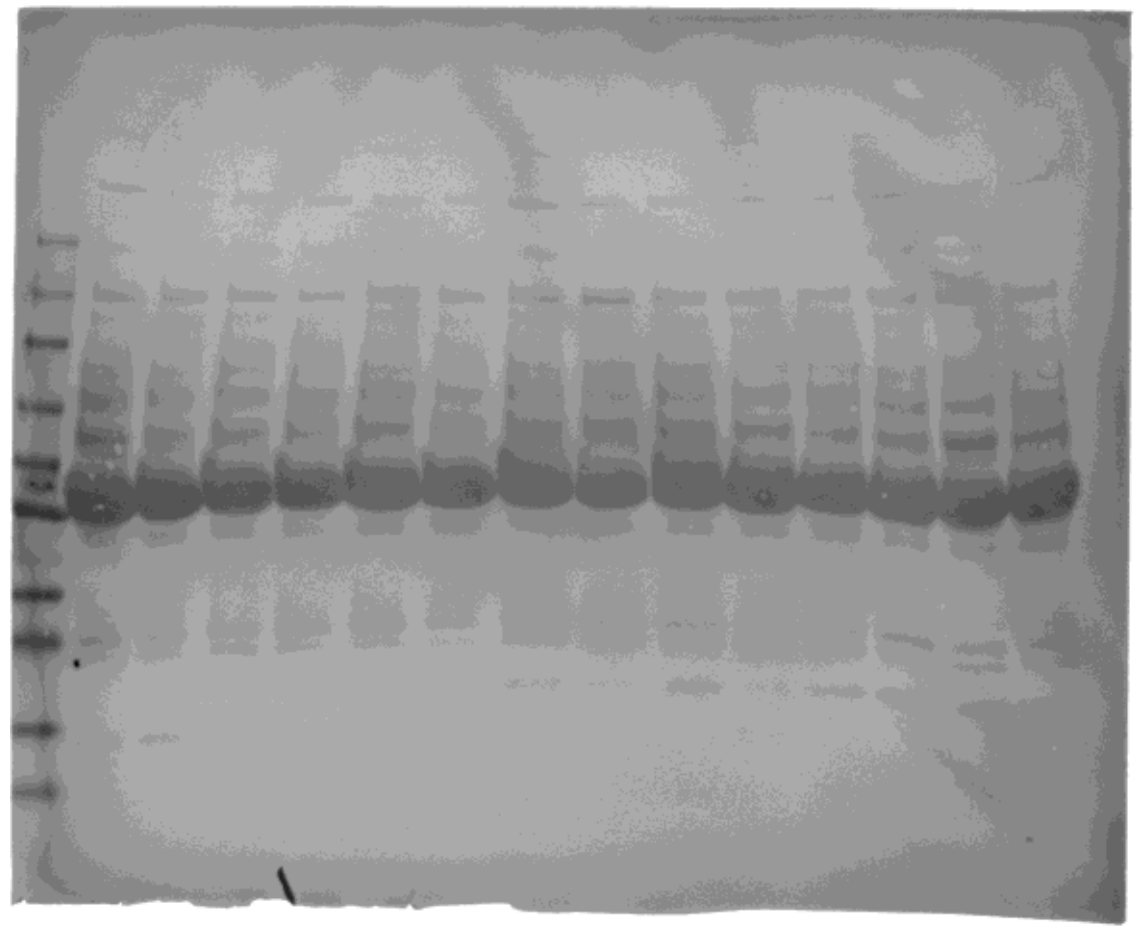

Western immunoblotting (whole membranes)

Gel 5 (samples 57-70)

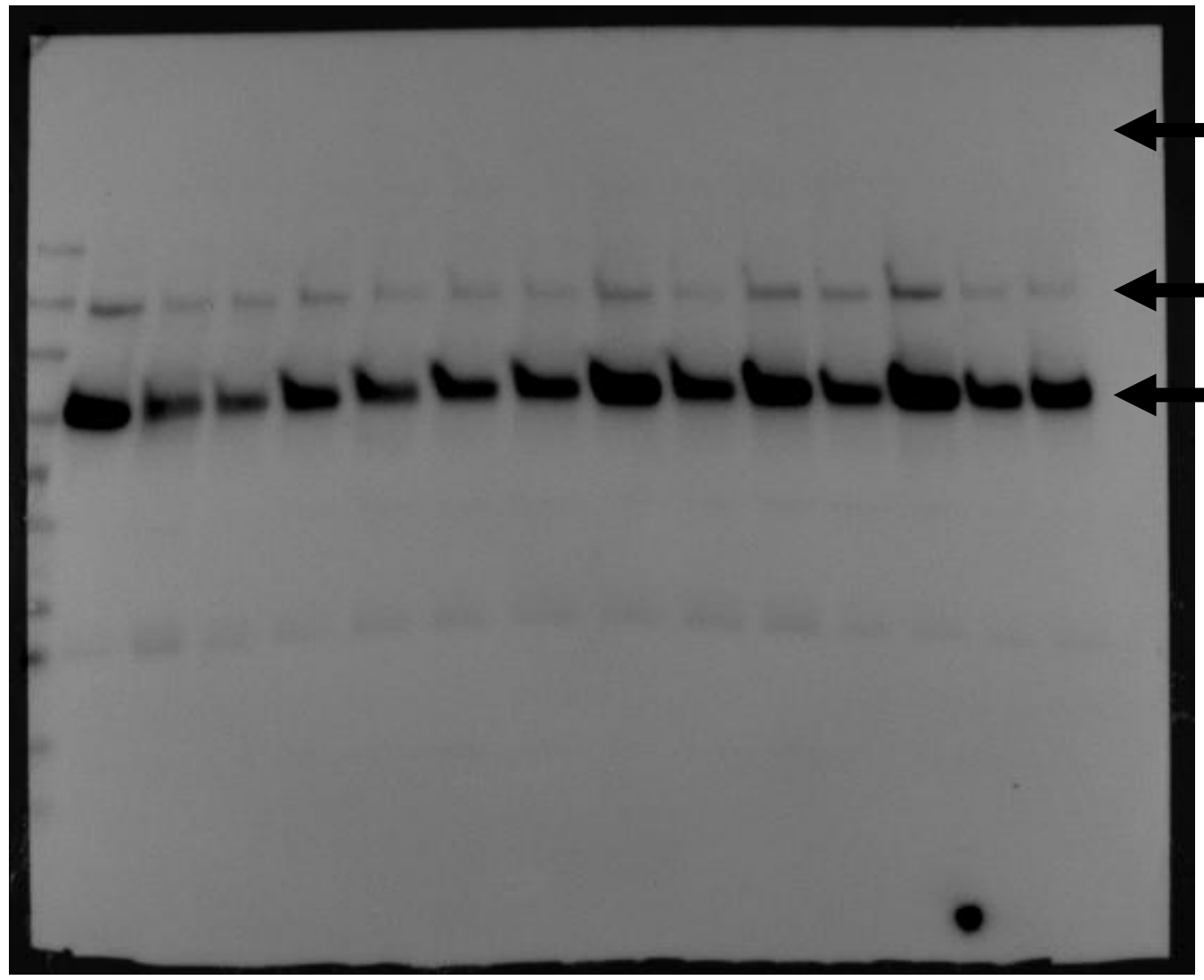

Gel 6 (samples 71-84)

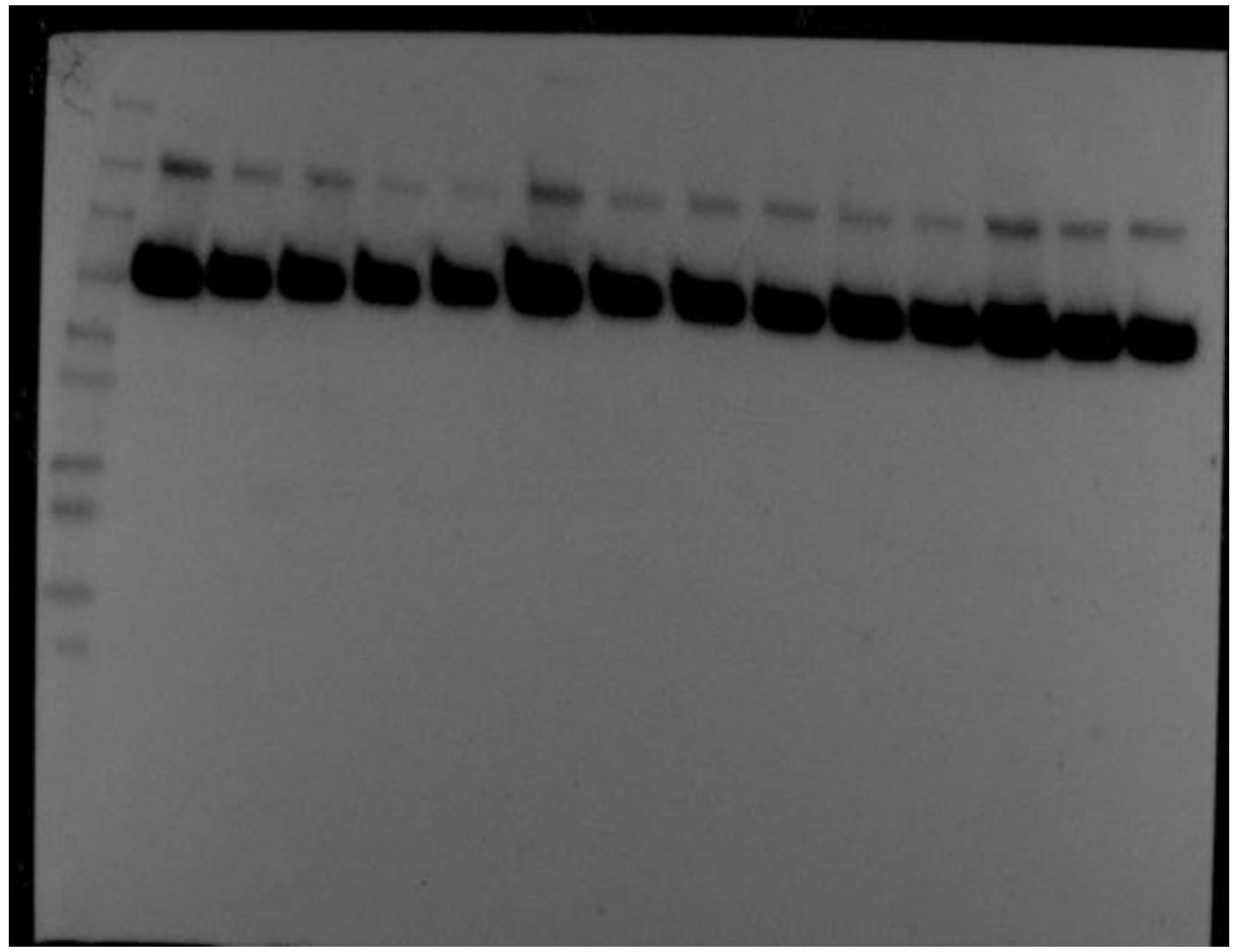

Ponceau 5

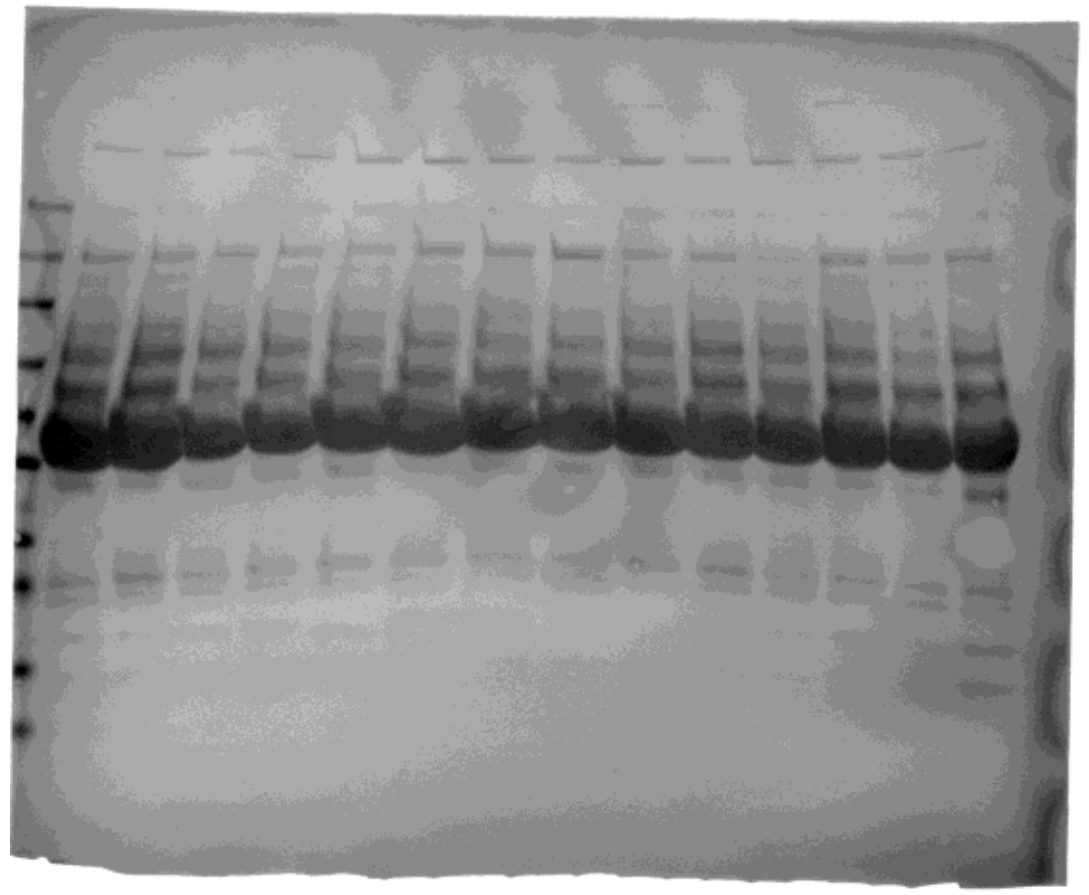

Ponceau 6

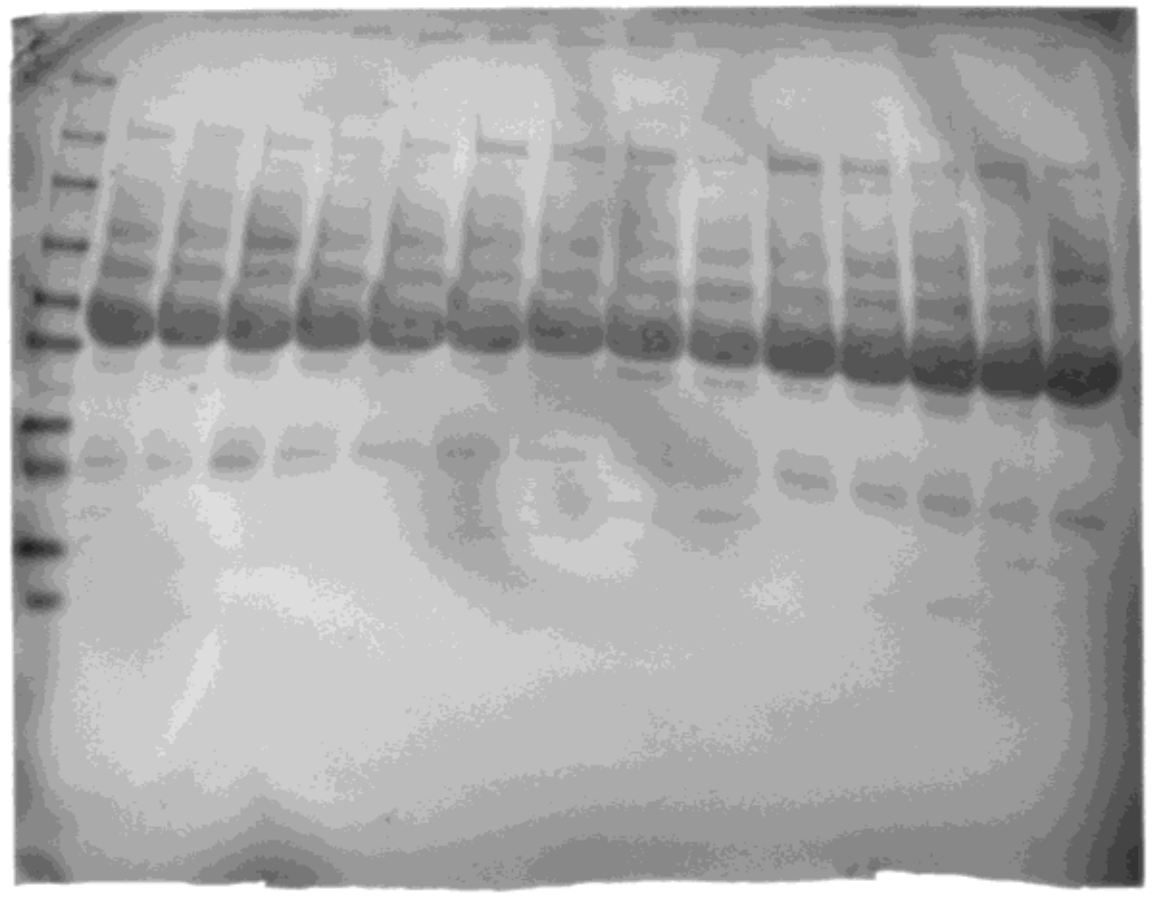

Western immunoblotting (whole membranes)

Gel 7 (samples 85-98)

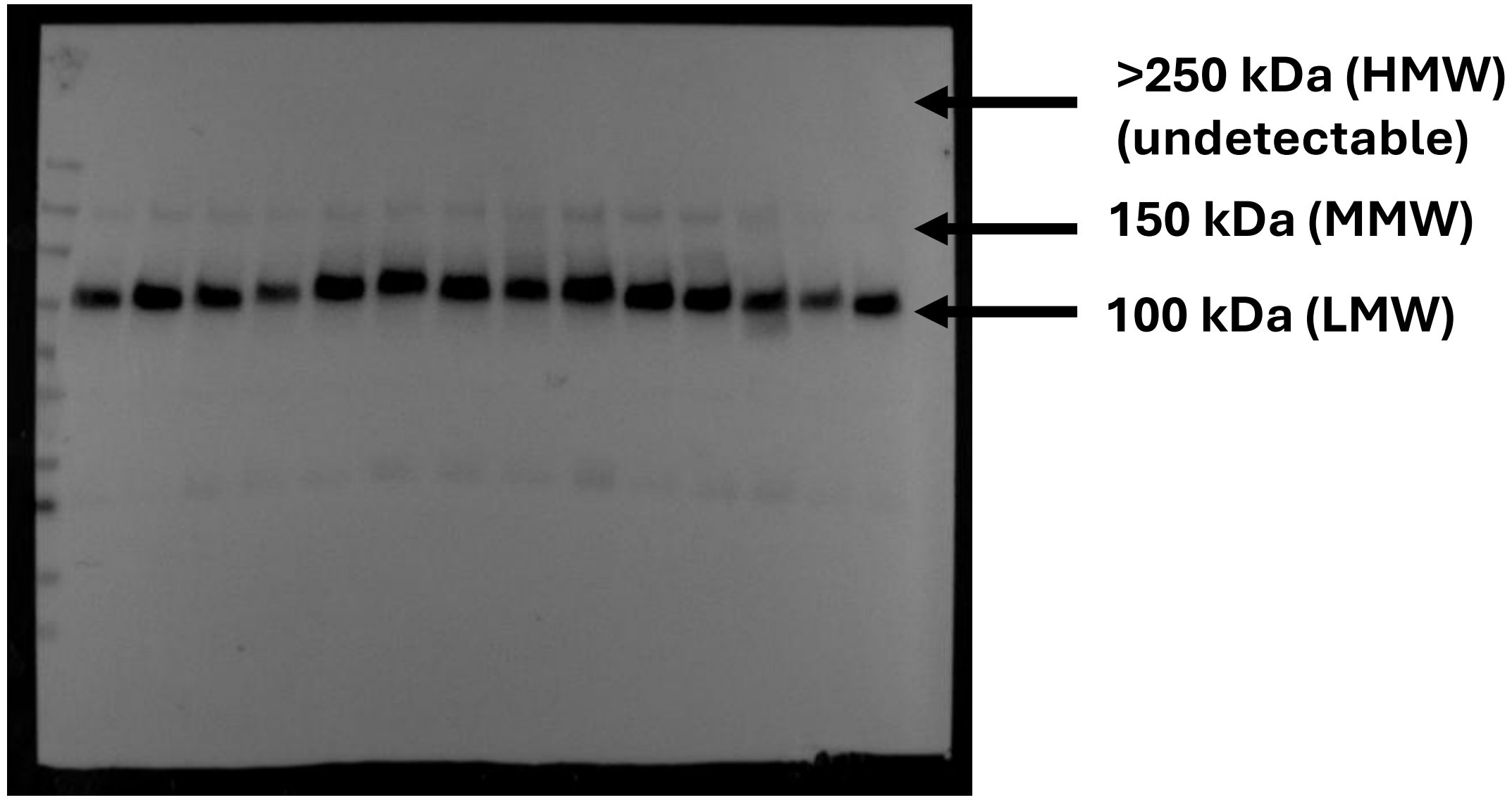

Gel 8 (samples 99-112)

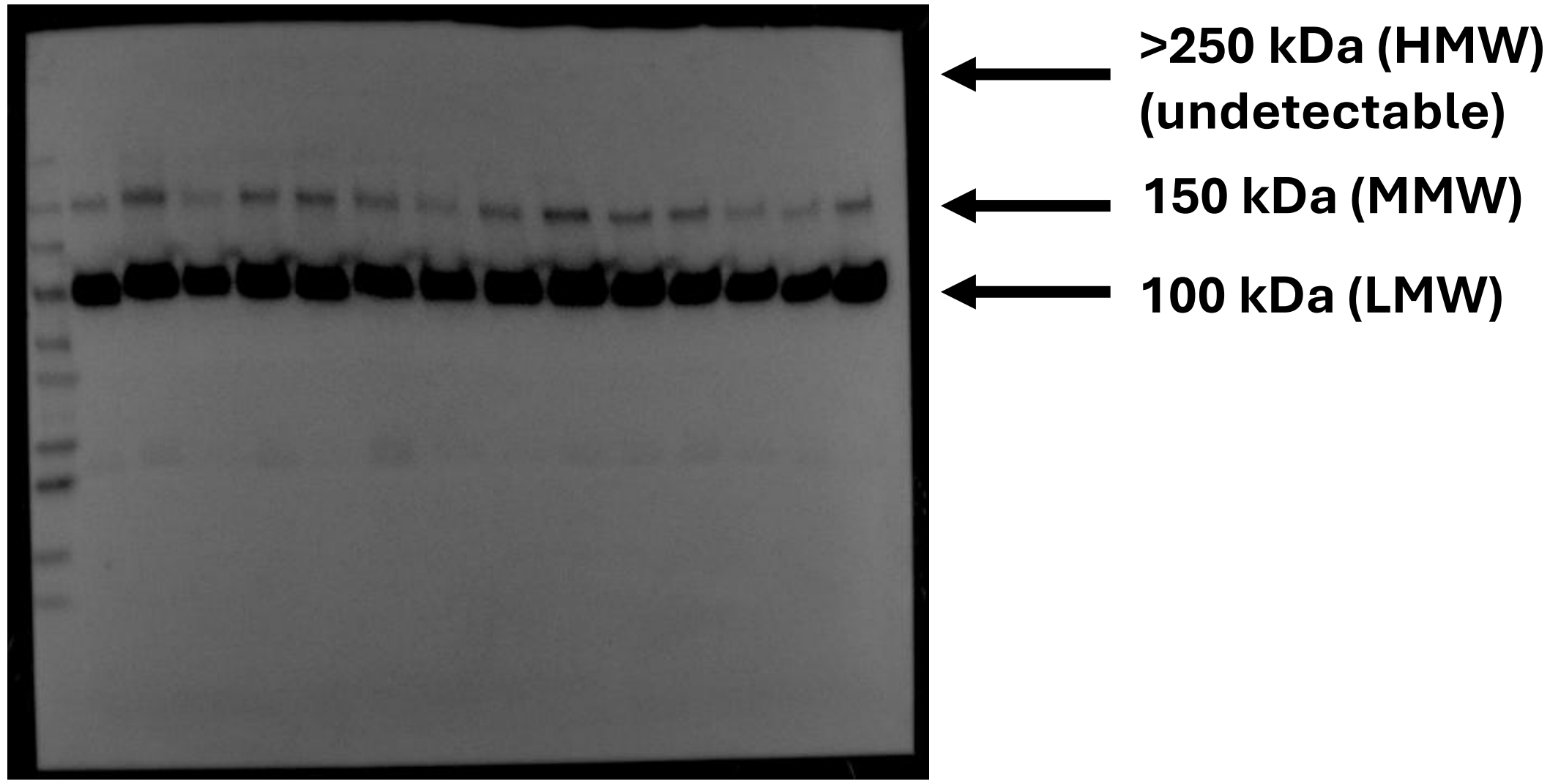

Ponceau 7

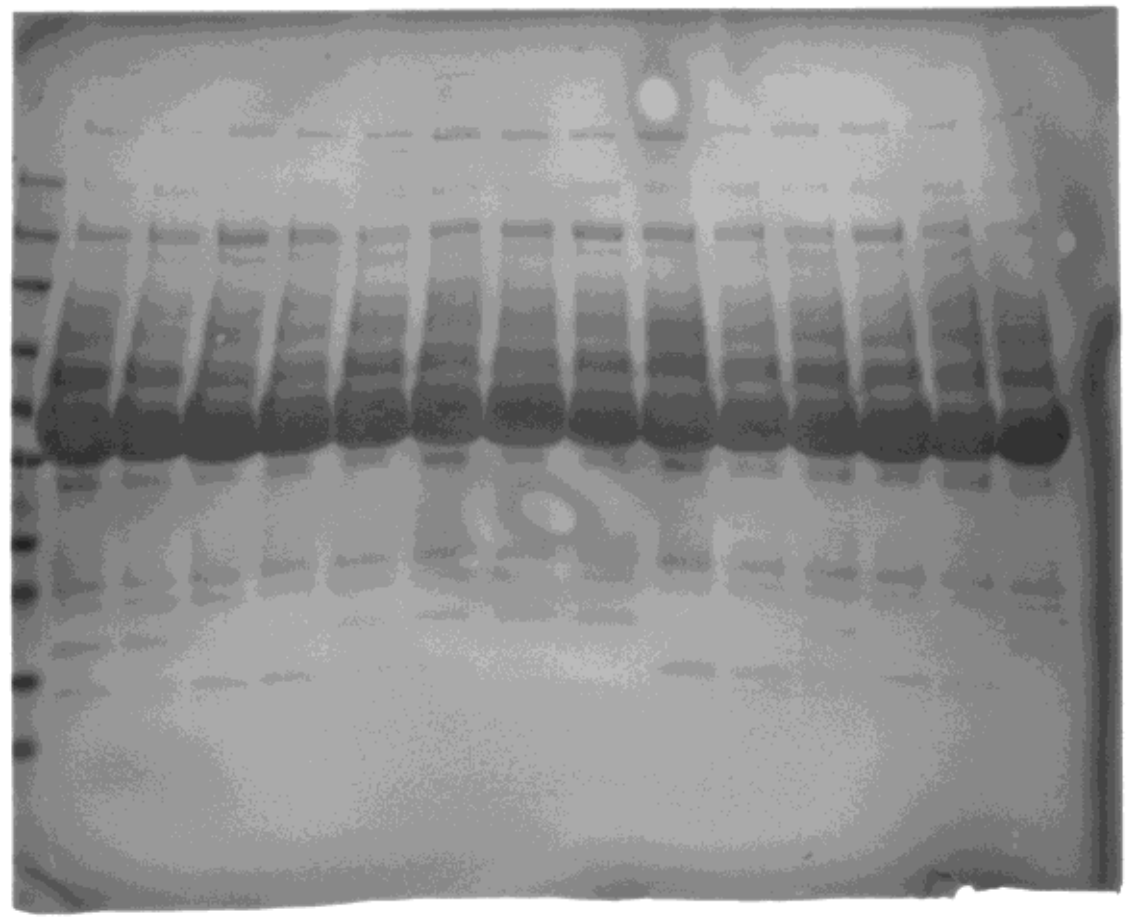

Ponceau 8

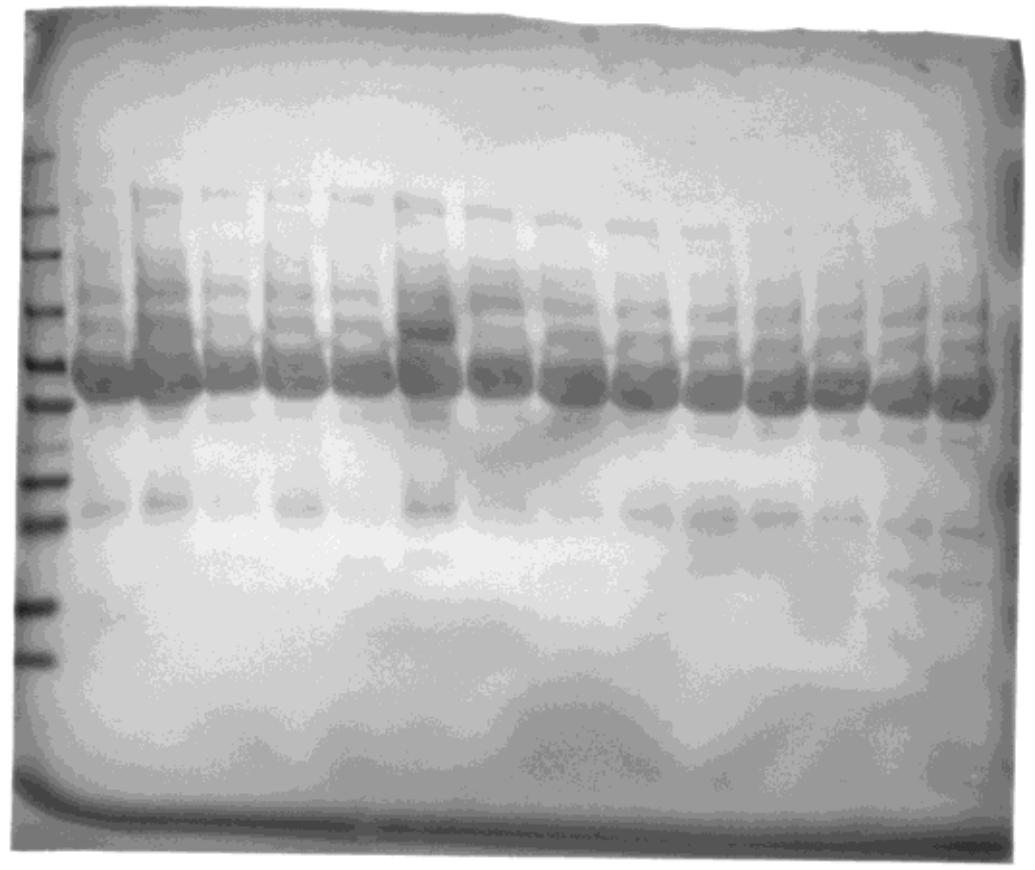

Supplement: Supplementary file 1 [file pone.0360041.s001.pdf]
