# Supplementary material for: Circulating low- and medium-molecular-weight adiponectin are associated with metabolic phenotypes in adults undergoing cardiometabolic screening
Source: PLoS One. 2026 Oct 5;21(10):e0360041. doi: 10.1371/journal.pone.0360041 (PMC13637893; doi:10.1371/journal.pone.0360041)
Supplement: Supplementary file 2 [file pone.0360041.s003.pdf]

Adiponectin is an adipokine that exerts insulin-sensitizing effects. In circulation, adiponectin exists in multimeric forms, including LMW, MMW, and HMW. However, the correlations between distinct multimeric forms and metabolic profile in humans remain unclear. Martínez-Huenchullán et al. measured circulating adiponectin multimers in overweight/obese patients and showed that MMW and the MMW/LMW ratio were inversely associated with adiposity, atherogenic lipid markers, and liver injury markers using Pearson correlation and PCA analysis. However, the data interpretation is insufficient to support the conclusions, and the manuscript needs more attention.

1) What were the numbers of men and women in the tables?
2) The description of what the parentheses mean in the tables is missing. This would make it hard for readers to understand the data.
3) What does MET stand for in physical activity? The authors referred to their previous paper in the methods section, but it would help to briefly define it.
4) The levels of liver injury markers, such as AST, ALT, and GGT, are within the normal range in the patients. The MMW adiponectin shows a negative correlation with these markers, but the significance is questionable.
5) The authors demonstrated that MMW adiponectin is inversely correlated with adiposity (line 264), but its correlation is stronger with lean mass (-0.33) than Fat mass (-0.16). In addition, the r value (coefficient) was -0.09 with ApoB. Therefore, it is questionable whether MMW adiponectin is negatively correlated with adiposity and ApoB.
6) No data supported the description in lines 267- 269.
7) Not sufficient explanation for Figure3. The description for Fig 3C, 3D, 3E, and 3F was missing.
8) In Figure 3F, there are no labels for each lane or a title.
