# Supplementary material for: Circulating low- and medium-molecular-weight adiponectin are associated with metabolic phenotypes in adults undergoing cardiometabolic screening
Source: PLoS One. 2026 Oct 5;21(10):e0360041. doi: 10.1371/journal.pone.0360041 (PMC13637893; doi:10.1371/journal.pone.0360041)
Supplement: Supplementary file 3 [file pone.0360041.s005.docx]

**Response to reviewers (round 1)**

**Manuscript: “Circulating low- and medium-molecular-weight adiponectin are associated with metabolic phenotypes in adults undergoing cardiometabolic screening”.**

Dear editor and reviewers,

Thank you for your comments and suggestions made to our manuscript. Undoubtedly, they improved the quality and clarity of our report. Please find our point-by-point reply to the editorial comment made.

**Journal requirements**

| **Comment** | **Location in manuscript** | **Reply** |
| --- | --- | --- |
| 1. Please ensure that your manuscript meets PLOS ONE's style requirements, including those for file naming. | Throughout | Thank you for your comment.  We updated the format of the revised manuscript to meet the journal’s requirements. |
| 2. Thank you for stating the following financial disclosure:  “This research was funded by the National Agency of Research and Development (ANID), FONDECYT de Iniciación N°11241108.”  Please state what role the funders took in the study. If the funders had no role, please state: "The funders had no role in study design, data collection and analysis, decision to publish, or preparation of the manuscript."  If this statement is not correct you must amend it as needed.  Please include this amended Role of Funder statement in your cover letter; we will change the online submission form on your behalf. | Funding | Thank you for your comment.  Since the funders did not have any role in the study, the appropriate text was added in this section. |
| 3. In the online submission form you indicate that your data is not available for proprietary reasons and have provided a contact point for accessing this data. Please note that your current contact point is a co-author on this manuscript. According to our Data Policy, the contact point must not be an author on the manuscript and must be an institutional contact, ideally not an individual. Please revise your data statement to a non-author institutional point of contact, such as a data access or ethics committee, and send this to us via return email. Please also include contact information for the third party organization, and please include the full citation of where the data can be found. |  | Thank you for this clarification. We have revised the Data Availability Statement so that data-access requests are directed to a non-author institutional contact rather than the corresponding author. |
| 4. Your ethics statement should only appear in the Methods section of your manuscript. If your ethics statement is written in any section besides the Methods, please delete it from any other section. | Methods | Thank you for your comment.  This information was only kept at the Methods section. |
| 5. PLOS ONE now requires that authors provide the original uncropped and unadjusted images underlying all blot or gel results reported in a submission’s figures or Supporting Information files. This policy and the journal’s other requirements for blot/gel reporting and figure preparation are described in detail at https://journals.plos.org/plosone/s/figures#loc-blot-and-gel-reporting-requirements and https://journals.plos.org/plosone/s/figures#loc-preparing-figures-from-image-files. When you submit your revised manuscript, please ensure that your figures adhere fully to these guidelines and provide the original underlying images for all blot or gel data reported in your submission. See the following link for instructions on providing the original image data: https://journals.plos.org/plosone/s/figures#loc-original-images-for-blots-and-gels.  In your cover letter, please note whether your blot/gel image data are in Supporting Information or posted at a public data repository, provide the repository URL if relevant, and provide specific details as to which raw blot/gel images, if any, are not available. Email us at plosone@plos.org if you have any questions. | Supporting information | Thank you for your comment.  We added a pdf file with the full blots of all the samples analyzed in this study as Supplementary information (S1_raw_images). |
| 6. Please include captions for your Supporting Information files at the end of your manuscript, and update any in-text citations to match accordingly. Please see our Supporting Information guidelines for more information: http://journals.plos.org/plosone/s/supporting-information.  If the reviewer comments include a recommendation to cite specific previously published works, please review and evaluate these publications to determine whether they are relevant and should be cited. There is no requirement to cite these works unless the editor has indicated otherwise. | Supporting information | Thank you for your comment.  We added this information at the end of the revised manuscript. |

**Reviewer 1**

| **Comment** | **Location in manuscript** | **Reply** |
| --- | --- | --- |
| Gel/membrane catalog number (Section 2.5). The manuscript cites Bio-Rad catalog no. 1704158 for both the acrylamide gradient gel and the nitrocellulose transfer membrane. This catalog number corresponds to the Trans-Blot Turbo Mini nitrocellulose transfer pack, not a gel product. Please confirm the correct catalog number and specify the actual acrylamide percentage/gradient range (e.g., 4–15%, 4–20%) used for the gel, as this detail is necessary for reproduction of the protocol. Please clarify whether this was a transcription error or whether a different gel product was in fact used. | Methods | Thank you for your comment.  We agree with you, it was a transcription error. Then correct catalog number is 4561086 which was amended in the revised manuscript.  The actual acrylamide gradient was 4-15%. |
| RIPA buffer and non-denaturing conditions. The samples are described as processed "under non-reducing and non-denaturing conditions," yet the loading mixture includes RIPA buffer, which conventionally contains ionic detergents (SDS, sodium deoxycholate) capable of disrupting non-covalent multimer assembly. Please specify the exact composition of the RIPA buffer used (standard formulation vs. a modified, detergent-reduced version) and the composition of the "6X loading buffer," and clarify how multimer integrity was confirmed to be preserved under these conditions. This is important for readers attempting to reproduce the assay, since a standard RIPA formulation would be expected to compromise the native multimeric structure the method is designed to preserve. | Methods | Thank you for your comments.  We agree with you that the RIPA and loading buffers have components that could disturbed some of the multimers. We acknowledge that conventional RIPA contains detergents that may affect non-covalent protein interactions and therefore agree that these conditions should not be described as strictly native. Nevertheless, the same sample-preparation and electrophoretic protocol was previously used by our group and successfully resolved LMW, MMW, and HMW adiponectin in human samples (Ref: Alvarado-Carrasco D, González-Maraboli A, Reyes-Montalva M, Videla-Aguilar J, Bentes A, Peñailillo L, Sandoval V, Martínez-Huenchullán S. Acute effects of high-intensity interval training on adiponectin isoforms in inactive young adults: a quasi-experimental study. BMC Sports Sci Med Rehabil. 2025 Dec 30;17(1):387. doi: 10.1186/s13102-025-01433-7), providing empirical evidence that the protocol can discriminate the three major multimeric forms.  Therefore, we are confident of the results presented here. We nevertheless acknowledge that protocol-related effects on multimer preservation cannot be completely excluded. |
| Serum input volume and electrophoresis voltage range (minor). Please confirm the rationale for the very small serum input volume (0.5 µL) and specify whether the reported electrophoresis range (80–120 V, ~60 min) reflects a ramped voltage protocol or a range across replicate runs. Precise, fixed parameters (or an explanation of the ramp) would improve reproducibility. | Methods | Thank you for your comment and suggestion.  We agree with you regarding that more detail in the methods are required.  The small volume of serum input was decided because of the protein abundancy in human serum. Higher serum volumes resulted in overloaded lanes, poorer electrophoretic separation, and unreliable densitometric quantification during protocol optimization This is one of our optimization runs with volumes from 1ul to 5 ul:    In terms of the voltage, we have specified that the first 10 minutes of the run the voltage was at 80 V, wheareas for the rest of the run was set at 120 V |
| I would ask the authors to confirm in their response letter whether points 1 and 3 reflect simple transcription errors, and to provide the corrected details for the record, since without this information the assay as described could not be reliably reproduced by another laboratory. | Methods | Thank you for your comments and suggestions.  We have addressed these points in the revised manuscript. |

**Reviewer 2**

| **Comment** | **Location in manuscript** | **Reply** |
| --- | --- | --- |
| 1) What were the numbers of men and women in the tables? | Results | Thank you for your comment.  We added the numbers of men and women in each table in the revised manuscript. |
| 2) The description of what the parentheses mean in the tables is missing. This would make it hard for readers to understand the data. | Results | Thank you for your comment.  We added the explanation of what the numbers and parentheses mean in the tables (mean and SD) in the revised manuscript. |
| 3) What does MET stand for in physical activity? The authors referred to their previous paper in the methods section, but it would help to briefly define it. | Methods and results | Thank you for your suggestion.  We have added the definition of MET (metabolic equivalent of task) in the revised manuscript. |
| 4) The levels of liver injury markers, such as AST, ALT, and GGT, are within the normal range in the patients. The MMW adiponectin shows a negative correlation with these markers, but the significance is questionable. | Results | Thank you for your comment.  We agree with you that the circulating AST, ALT, and GGT values in our cohort were, on average, within commonly accepted clinical reference ranges. Therefore, we agree that the observed inverse associations between MMW adiponectin and these enzymes should not be interpreted as evidence of liver injury or clinically relevant hepatic dysfunction. Our intention was instead to describe associations across the continuous range of liver-enzyme values observed in this metabolically heterogeneous cohort. Variation in these enzymes within the reference range may still accompany differences in metabolic phenotype; however, given the modest magnitude of the correlations and the cross-sectional and exploratory nature of our study, their clinical significance remains uncertain. We have therefore revised the manuscript to avoid referring to these findings as evidence of liver injury or hepatic risk and now describe them more cautiously as associations with liver-enzyme levels. We have also emphasized that these findings should be considered hypothesis-generating and require confirmation in studies incorporating direct measures of hepatic steatosis or liver function. |
| 5) The authors demonstrated that MMW adiponectin is inversely correlated with adiposity (line 264), but its correlation is stronger with lean mass (-0.33) than Fat mass (-0.16). In addition, the r value (coefficient) was -0.09 with ApoB. Therefore, it is questionable whether MMW adiponectin is negatively correlated with adiposity and ApoB. | Results | We thank the reviewer for this careful observation and agree that our original wording was too broad. Specifically, the correlation between MMW adiponectin and fat mass was weak (r = -0.16), whereas the association with lean mass was stronger (r = -0.33). Because lean mass should not be interpreted as a marker of adiposity, we have revised the manuscript to avoid describing this pattern generically as an inverse association with adiposity. We now refer more cautiously to associations with selected anthropometric and body-composition variables.  We also agree that the correlation between MMW adiponectin and ApoB was very small (r = -0.09) and does not support a meaningful inverse association. We have therefore removed ApoB from statements describing the direct correlates of MMW adiponectin. Where relevant, ApoB is retained only in analyses in which it contributes to a broader multivariate pattern, such as the PCA, and this is now clearly distinguished from a direct bivariate association. These revisions provide a more accurate representation of the magnitude and specificity of the observed associations. |
| 6) No data supported the description in lines 267- 269. | Results | Thank you for your comment.  We have updated the manuscript accordingly. |
| 7) Not sufficient explanation for Figure3. The description for Fig 3C, 3D, 3E, and 3F was missing. | Results | We thank the reviewer for this comment. We agree that the original Figure 3 legend did not provide sufficient information for all panels. We have therefore expanded the legend to explicitly describe panels C–F, including the criteria used to define low HDL-C (<40 mg/dL), high triglycerides (≥150 mg/dL), and high fasting glycemia (≥100 mg/dL), as well as the content of the representative Western blot shown in panel F. |
| 8) In Figure 3F, there are no labels for each lane or a title. | Results | We thank the reviewer for identifying this lack of clarity. Panel F presents a representative Western immunoblot and was not intended to represent the categorical comparisons shown in panels A–E. We have now added a descriptive title to panel F and labeled the individual lanes. We have also clarified in the figure legend that each lane corresponds to serum from an individual participant and that the blot is provided as a representative example of LMW and MMW adiponectin detection rather than as a direct visualization of the group comparisons. |

**Reviewer 3**

| **Comment** | **Location in manuscript** | **Reply** |
| --- | --- | --- |
| 1. Definition of the analytic population.  The eligibility criteria state a BMI of 25.0–34.9 kg/m² and fasting triglycerides of 100–215 mg/dL. However, the descriptive statistics appear incompatible with these restrictions, particularly triglycerides (140 ± 76.2 mg/dL). Figure 1 also indicates that the 112 participants “completed the screening visit,” rather than showing that all 112 satisfied the eligibility criteria. Please clarify whether the current analysis included all participants completing screening or only those ultimately eligible for the parent RIO trial. The actual ranges of BMI and triglycerides and the numbers excluded at each eligibility step should be reported. If screening participants outside the parent trial eligibility range were analyzed, the description of the study population should be revised throughout the manuscript. | Throughout the manuscript | Thank you for your comment.  Yes, as you mentioned, all participants completing the screening process were included in these analyses. Therefore, not all of them were included in the clinical trial as they did not fulfill the eligibility criteria. Therefore, we clarified this point throughout the manuscript.  We have revised the Methods to clearly distinguish the screening cohort used in the present study from the subset subsequently enrolled in the RIO trial. We have also revised Figure 1 to show the progression from the 112 screened participants to the 40 participants enrolled in the intervention phase, including the reasons and numbers for exclusion at each eligibility step. In addition, we now report the actual observed ranges of BMI (21.9–46.1 kg/m²) and triglycerides (38–347 mg/dL) among the 112 participants included in the cross-sectional analysis. Finally, the Abstract and relevant sections of the manuscript have been revised to avoid implying that all 112 participants satisfied the parent trial eligibility criteria. |
| 2. Claims regarding total adiponectin and multimer distribution.  The manuscript repeatedly concludes that multimer measurements provide information “beyond total adiponectin concentration.” However, total adiponectin was not measured, and HMW adiponectin was not detectable. Thus, the current study cannot formally demonstrate incremental information beyond total adiponectin or characterize the complete adiponectin multimer distribution. The authors should either provide a direct comparison with total adiponectin, if available, or revise these claims throughout the Abstract, Key Points, Discussion, and Conclusion. The title should also more precisely reflect that only LMW and MMW adiponectin were quantified. In addition, because the measurements are normalized Western blot signals expressed in arbitrary units, terms implying absolute “concentrations” should be avoided. | Throughout the manuscript | We thank the reviewer for this important comment and agree with the concerns raised. Total adiponectin was not measured in the present study, and HMW adiponectin was not consistently detectable under our experimental conditions. Therefore, our data do not allow us to demonstrate that LMW or MMW adiponectin provide incremental information beyond total adiponectin, nor do they permit characterization of the complete circulating adiponectin multimer distribution.  We have therefore revised the manuscript throughout to remove claims that our findings provide information “beyond total adiponectin concentration.” We now restrict our interpretation to the distinct association patterns observed for the two adiponectin multimers that were quantified, LMW and MMW, and their MMW/LMW ratio. We have also revised terminology referring to the “complete multimer distribution” and now use more precise expressions such as “LMW and MMW adiponectin profile,” “relative LMW–MMW pattern,” or “MMW/LMW ratio,” as appropriate.  In addition, because adiponectin was quantified as Western blot band intensity normalized to total lane protein, we agree that the term “concentration” is inappropriate. We have replaced this terminology with “normalized signal,” “relative abundance,” or “adiponectin level” where appropriate and clarify that all Western blot-derived values are expressed in arbitrary units.  Finally, we have revised the title to explicitly reflect that the study quantified LMW and MMW adiponectin rather than the complete adiponectin multimer distribution. |
| 3. Potential confounding of the main associations.  The principal correlation analyses are unadjusted despite substantial sex differences in both adiponectin measures and several metabolic variables. Women showed higher MMW adiponectin and MMW/LMW ratios as well as a more favorable lipid profile. Therefore, some of the observed associations may be driven by sex rather than representing independent relationships. I recommend multivariable analyses for the main continuous associations using clearly justified covariate sets, including age, sex, smoking, and relevant adiposity measures where appropriate. Sex-stratified sensitivity analyses may also be informative. The authors should additionally report potentially relevant medication use, particularly lipid-lowering therapy. | Methods and results | We thank the reviewer for this important observation and agree that sex may confound several of the unadjusted associations, given the substantial sex differences observed in both adiponectin measures and cardiometabolic variables. In response, we performed additional multivariable linear regression analyses of the principal continuous associations.  Because MMW adiponectin and the MMW/LMW ratio showed right-skewed distributions, these outcomes were natural-log transformed before analysis. Models examining anthropometric variables were adjusted for age, sex, and smoking status, whereas models examining biochemical and metabolic variables were additionally adjusted for BMI. Continuous variables were standardized, and heteroskedasticity-robust (HC3) standard errors were used.  The additional analyses showed that several associations observed in the unadjusted correlations were attenuated after covariate adjustment, including those involving BMI/body-composition variables, triglycerides, VLDL-C, HDL-C, ApoB, and uric acid. In contrast, MMW adiponectin remained inversely associated with GGT (standardized β = −0.37, 95% CI −0.58 to −0.16, p<0.001) and ALT (β = −0.32, 95% CI −0.52 to −0.11, p=0.002), while the association with AST was borderline (β = −0.20, p=0.054). Similarly, the MMW/LMW ratio remained inversely associated with GGT (β = −0.36, p=0.004) and ALT (β = −0.25, p=0.009).  We also conducted sex-stratified sensitivity analyses. Associations with GGT and ALT tended to be stronger among women; however, formal predictor-by-sex interaction tests were not statistically significant. We therefore do not interpret these findings as evidence of sex-specific associations.  Regarding medication use, this information was unfortunately not systematically collected during the screening stage from which the present cross-sectional dataset was derived. We therefore cannot appropriately adjust for lipid-lowering medication use or assume its absence. We have now explicitly acknowledged this limitation in the revised manuscript. |
| 4. Multiple comparisons.  A large number of correlations and categorical comparisons were performed using a nominal P<0.05 threshold. Given the exploratory nature and number of tests, the risk of false-positive findings is substantial. I recommend controlling the false discovery rate, for example using the Benjamini–Hochberg procedure, and reporting adjusted P values. Alternatively, the authors should clearly distinguish prespecified primary hypotheses from exploratory analyses and temper conclusions based on nominal statistical significance. | Methods and Results | We thank the reviewer for this important comment and agree that the number of comparisons performed increases the risk of false-positive findings. In response, we applied the Benjamini–Hochberg false discovery rate (FDR) procedure to the principal multivariable continuous-association analyses and report FDR-adjusted q values in Supplementary Table S2. Following FDR correction, the inverse associations of MMW adiponectin with GGT and ALT and of the MMW/LMW ratio with GGT remained statistically significant, whereas several associations identified in the unadjusted exploratory correlations did not remain supported after multivariable adjustment and/or FDR correction.  We have therefore revised the manuscript to clearly distinguish the exploratory Pearson correlation analysis from the multivariable analyses used for inferential interpretation. The correlation matrix is retained as an exploratory visualization of the overall association pattern, whereas conclusions are now based primarily on associations that remained supported after covariate adjustment and FDR correction.  We additionally applied Benjamini–Hochberg FDR correction to the categorical phenotype comparisons shown in Figure 3. |
| 5. PCA methodology.  The PCA includes LMW, MMW, and the mathematically derived MMW/LMW ratio simultaneously. This redundancy may contribute to artificial component structure. Additional redundancy is likely among several closely related lipid measures. The PCA should therefore be repeated using a non-redundant variable set, and the robustness of the main findings should be demonstrated. Please also specify whether variables were standardized, whether a correlation or covariance matrix was used, how missing values were handled, and whether rotation was applied. Consider using parallel analysis in addition to the eigenvalue >1 criterion. Finally, PCA identifies components rather than participant “clusters,” and the corresponding terminology should be corrected. | Throughout the manuscript | We thank the reviewer for this important methodological comment. We repeated the PCA using a reduced, non-redundant variable set, excluding the mathematically derived MMW/LMW ratio and removing highly overlapping anthropometric and lipid variables. PCA was performed using the correlation matrix with listwise deletion of missing values (n=104). Because PCA was based on the correlation matrix, variables were standardized with respect to their variance. Missing values were handled using listwise deletion (n=104), and no rotation was applied. Five components had eigenvalues >1 and together explained 66.6% of the total variance. Because our aim was to preserve the original principal-component structure for exploratory interpretation rather than optimize a rotated factor solution, no rotation was applied. We also considered the reviewer's suggestion regarding parallel analysis. Because the purpose of the present PCA was explicitly exploratory and aimed to preserve potentially informative secondary dimensions of covariation rather than establish a definitive latent dimensional structure, we retained the eigenvalue >1 criterion. We have made this rationale explicit and now interpret the resulting components cautiously as exploratory, hypothesis-generating patterns rather than validated latent constructs.  We have clarified this in the Methods and Figure 4 legend. We have also replaced the term “clusters” with “components” throughout the manuscript. |

Sincerely,

**Sergio Martínez-Huenchullán, on behalf of all co-authors**

**Corresponding author**

**sergio.martinez@uss.cl**
