# Supplementary material for: Circulating low- and medium-molecular-weight adiponectin are associated with metabolic phenotypes in adults undergoing cardiometabolic screening
Source: PLoS One. 2026 Oct 5;21(10):e0360041. doi: 10.1371/journal.pone.0360041 (PMC13637893; doi:10.1371/journal.pone.0360041)
Supplement: Peer review history

# Peer review: Decision Letter 0

**DOI:** 10.1371/journal.pone.0360041.r001

**Authors:** Etsuro Ito

3 Sep 2026

Dear Dr. Sandoval,

Please submit your revised manuscript by Oct 17 2026 11:59PM. If you will need more time than this to complete your revisions, please reply to this message or contact the journal office at plosone@plos.org. When you're ready to submit your revision, log on to https://www.editorialmanager.com/pone/ and select the 'Submissions Needing Revision' folder to locate your manuscript file.

- A letter that responds to each point raised by the academic editor and reviewer(s). You should upload this letter as a separate file labeled 'Response to Reviewers'.
- A marked-up copy of your manuscript that highlights changes made to the original version. You should upload this as a separate file labeled 'Revised Manuscript with Track Changes'.
- An unmarked version of your revised paper without tracked changes. You should upload this as a separate file labeled 'Manuscript'.

If you would like to make changes to your financial disclosure, please include your updated statement in your cover letter. Guidelines for resubmitting your figure files are available below the reviewer comments at the end of this letter.

If applicable, we recommend that you deposit your laboratory protocols in protocols.io to enhance the reproducibility of your results. Protocols.io assigns your protocol its own identifier (DOI) so that it can be cited independently in the future. For instructions see: https://journals.plos.org/plosone/s/submission-guidelines#loc-laboratory-protocols. Additionally, PLOS One offers an option for publishing peer-reviewed Lab Protocol articles, which describe protocols hosted on protocols.io. Read more information on sharing protocols at https://plos.org/protocols?utm_medium=editorial-email&utm_source=authorletters&utm_campaign=protocols.

As the corresponding author, your ORCID iD is verified in the submission system and will appear in the published article. PLOS supports the use of ORCID, and we encourage all coauthors to register for an ORCID iD and use it as well. Please encourage your coauthors to verify their ORCID iD within the submission system before final acceptance, as unverified ORCID iDs will not appear in the published article. *Only*  the individual author can complete the verification step; PLOS staff *cannot*  verify ORCID iDs on behalf of authors.

We look forward to receiving your revised manuscript.

Kind regards,

Etsuro Ito, Ph.D.

Academic Editor

PLOS One

Journal requirements:

When submitting your revision, we need you to address these additional requirements.

1. Please ensure that your manuscript meets PLOS ONE's style requirements, including those for file naming. The PLOS ONE style templates can be found at

https://journals.plos.org/plosone/s/file?id=wjVg/PLOSOne_formatting_sample_main_body.pdf and

https://journals.plos.org/plosone/s/file?id=ba62/PLOSOne_formatting_sample_title_authors_affiliations.pdf

2. Thank you for stating the following financial disclosure:

“This research was funded by the National Agency of Research and Development (ANID), FONDECYT de Iniciación N°11241108.”

Please state what role the funders took in the study.  If the funders had no role, please state: "The funders had no role in study design, data collection and analysis, decision to publish, or preparation of the manuscript."

If this statement is not correct you must amend it as needed.

Please include this amended Role of Funder statement in your cover letter; we will change the online submission form on your behalf.

3. In the online submission form you indicate that your data is not available for proprietary reasons and have provided a contact point for accessing this data. Please note that your current contact point is a co-author on this manuscript. According to our Data Policy, the contact point must not be an author on the manuscript and must be an institutional contact, ideally not an individual. Please revise your data statement to a non-author institutional point of contact, such as a data access or ethics committee, and send this to us via return email. Please also include contact information for the third party organization, and please include the full citation of where the data can be found.

4. Your ethics statement should only appear in the Methods section of your manuscript. If your ethics statement is written in any section besides the Methods, please delete it from any other section.

5. PLOS ONE now requires that authors provide the original uncropped and unadjusted images underlying all blot or gel results reported in a submission’s figures or Supporting Information files. This policy and the journal’s other requirements for blot/gel reporting and figure preparation are described in detail at https://journals.plos.org/plosone/s/figures#loc-blot-and-gel-reporting-requirements and https://journals.plos.org/plosone/s/figures#loc-preparing-figures-from-image-files. When you submit your revised manuscript, please ensure that your figures adhere fully to these guidelines and provide the original underlying images for all blot or gel data reported in your submission. See the following link for instructions on providing the original image data: https://journals.plos.org/plosone/s/figures#loc-original-images-for-blots-and-gels.

In your cover letter, please note whether your blot/gel image data are in Supporting Information or posted at a public data repository, provide the repository URL if relevant, and provide specific details as to which raw blot/gel images, if any, are not available. Email us at plosone@plos.org if you have any questions.

6. Please include captions for your Supporting Information files at the end of your manuscript, and update any in-text citations to match accordingly. Please see our Supporting Information guidelines for more information: http://journals.plos.org/plosone/s/supporting-information.

If the reviewer comments include a recommendation to cite specific previously published works, please review and evaluate these publications to determine whether they are relevant and should be cited. There is no requirement to cite these works unless the editor has indicated otherwise.

Reviewers' comments:

Reviewer's Responses to Questions

**Comments to the Author**

1. Is the manuscript technically sound, and do the data support the conclusions?

Reviewer #1: Yes

Reviewer #2: Partly

Reviewer #3: Yes

**********

2. Has the statistical analysis been performed appropriately and rigorously?

Reviewer #1: Yes

Reviewer #2: Yes

Reviewer #3: Yes

**********

3. Have the authors made all data underlying the findings in their manuscript fully available?

The PLOS Data policy

Reviewer #1: Yes

Reviewer #2: No

Reviewer #3: Yes

**********

4. Is the manuscript presented in an intelligible fashion and written in standard English?

Reviewer #1: Yes

Reviewer #2: Yes

Reviewer #3: Yes

**********

Reviewer #1: Overall Assessment:

This is a well-conducted, carefully analyzed cross-sectional study addressing a genuinely important gap: the tendency of the field to rely on total adiponectin rather than multimer-specific measures. The statistical approach (correlation, adjusted regression, and PCA used complementarily rather than in isolation) is appropriate and the authors are appropriately cautious in their causal language given the cross-sectional design. The chrononutrition angle adds a novel dimension not previously explored in this context. I find the manuscript scientifically sound and suitable for publication, subject to the authors clarifying the methodological points below, which bear on reproducibility of the Western blotting protocol.

Comments to Authors:

Gel/membrane catalog number (Section 2.5). The manuscript cites Bio-Rad catalog no. 1704158 for both the acrylamide gradient gel and the nitrocellulose transfer membrane. This catalog number corresponds to the Trans-Blot Turbo Mini nitrocellulose transfer pack, not a gel product. Please confirm the correct catalog number and specify the actual acrylamide percentage/gradient range (e.g., 4–15%, 4–20%) used for the gel, as this detail is necessary for reproduction of the protocol. Please clarify whether this was a transcription error or whether a different gel product was in fact used.

RIPA buffer and non-denaturing conditions. The samples are described as processed "under non-reducing and non-denaturing conditions," yet the loading mixture includes RIPA buffer, which conventionally contains ionic detergents (SDS, sodium deoxycholate) capable of disrupting non-covalent multimer assembly. Please specify the exact composition of the RIPA buffer used (standard formulation vs. a modified, detergent-reduced version) and the composition of the "6X loading buffer," and clarify how multimer integrity was confirmed to be preserved under these conditions. This is important for readers attempting to reproduce the assay, since a standard RIPA formulation would be expected to compromise the native multimeric structure the method is designed to preserve.

Serum input volume and electrophoresis voltage range (minor). Please confirm the rationale for the very small serum input volume (0.5 µL) and specify whether the reported electrophoresis range (80–120 V, ~60 min) reflects a ramped voltage protocol or a range across replicate runs. Precise, fixed parameters (or an explanation of the ramp) would improve reproducibility.

I would ask the authors to confirm in their response letter whether points 1 and 3 reflect simple transcription errors, and to provide the corrected details for the record, since without this information the assay as described could not be reliably reproduced by another laboratory.

Recommendation: Minor revision — the study is otherwise sound and I support publication once the authors address the methodological clarifications above.

Reviewer #2: Adiponectin is an adipokine that exerts insulin-sensitizing effects. In circulation, adiponectin exists in multimeric forms, including LMW, MMW, and HMW. However, the correlations between distinct multimeric forms and metabolic profile in humans remain unclear. Martínez-Huenchullán et al. measured circulating adiponectin multimers in overweight/obese patients and showed that MMW and the MMW/LMW ratio were inversely associated with adiposity, atherogenic lipid markers, and liver injury markers using Pearson correlation and PCA analysis. However, the data interpretation is insufficient to support the conclusions, and the manuscript needs more attention.

1) What were the numbers of men and women in the tables?

2) The description of what the parentheses mean in the tables is missing. This would make it hard for readers to understand the data.

3) What does MET stand for in physical activity? The authors referred to their previous paper in the methods section, but it would help to briefly define it.

4) The levels of liver injury markers, such as AST, ALT, and GGT, are within the normal range in the patients. The MMW adiponectin shows a negative correlation with these markers, but the significance is questionable.

5) The authors demonstrated that MMW adiponectin is inversely correlated with adiposity (line 264), but its correlation is stronger with lean mass (-0.33) than Fat mass (-0.16). In addition, the r value (coefficient) was -0.09 with ApoB. Therefore, it is questionable whether MMW adiponectin is negatively correlated with adiposity and ApoB.

6) No data supported the description in lines 267- 269.

7) Not sufficient explanation for Figure3. The description for Fig 3C, 3D, 3E, and 3F was missing.

8) In Figure 3F, there are no labels for each lane or a title.

Reviewer #3: This cross-sectional study examines the associations of circulating LMW and MMW adiponectin and the MMW/LMW ratio with metabolic, behavioral, and chrononutrition-related phenotypes in 112 adults. The topic is potentially interesting, and the integration of adiponectin multimer measurements with extensive metabolic phenotyping is a strength. The manuscript is generally clearly written. However, several methodological and statistical issues currently limit the interpretation of the findings. In particular, clarification of the analytic population, additional adjustment for important confounders, consideration of multiple testing, and revision of the PCA and Western blot methodology are needed. I therefore believe that substantial revision is required before the conclusions can be adequately supported.

1. Definition of the analytic population.

The eligibility criteria state a BMI of 25.0–34.9 kg/m² and fasting triglycerides of 100–215 mg/dL. However, the descriptive statistics appear incompatible with these restrictions, particularly triglycerides (140 ± 76.2 mg/dL). Figure 1 also indicates that the 112 participants “completed the screening visit,” rather than showing that all 112 satisfied the eligibility criteria. Please clarify whether the current analysis included all participants completing screening or only those ultimately eligible for the parent RIO trial. The actual ranges of BMI and triglycerides and the numbers excluded at each eligibility step should be reported. If screening participants outside the parent trial eligibility range were analyzed, the description of the study population should be revised throughout the manuscript.

2. Claims regarding total adiponectin and multimer distribution.

The manuscript repeatedly concludes that multimer measurements provide information “beyond total adiponectin concentration.” However, total adiponectin was not measured, and HMW adiponectin was not detectable. Thus, the current study cannot formally demonstrate incremental information beyond total adiponectin or characterize the complete adiponectin multimer distribution. The authors should either provide a direct comparison with total adiponectin, if available, or revise these claims throughout the Abstract, Key Points, Discussion, and Conclusion. The title should also more precisely reflect that only LMW and MMW adiponectin were quantified. In addition, because the measurements are normalized Western blot signals expressed in arbitrary units, terms implying absolute “concentrations” should be avoided.

3. Potential confounding of the main associations.

The principal correlation analyses are unadjusted despite substantial sex differences in both adiponectin measures and several metabolic variables. Women showed higher MMW adiponectin and MMW/LMW ratios as well as a more favorable lipid profile. Therefore, some of the observed associations may be driven by sex rather than representing independent relationships. I recommend multivariable analyses for the main continuous associations using clearly justified covariate sets, including age, sex, smoking, and relevant adiposity measures where appropriate. Sex-stratified sensitivity analyses may also be informative. The authors should additionally report potentially relevant medication use, particularly lipid-lowering therapy.

4. Multiple comparisons.

A large number of correlations and categorical comparisons were performed using a nominal P<0.05 threshold. Given the exploratory nature and number of tests, the risk of false-positive findings is substantial. I recommend controlling the false discovery rate, for example using the Benjamini–Hochberg procedure, and reporting adjusted P values. Alternatively, the authors should clearly distinguish prespecified primary hypotheses from exploratory analyses and temper conclusions based on nominal statistical significance.

5. PCA methodology.

The PCA includes LMW, MMW, and the mathematically derived MMW/LMW ratio simultaneously. This redundancy may contribute to artificial component structure. Additional redundancy is likely among several closely related lipid measures. The PCA should therefore be repeated using a non-redundant variable set, and the robustness of the main findings should be demonstrated. Please also specify whether variables were standardized, whether a correlation or covariance matrix was used, how missing values were handled, and whether rotation was applied. Consider using parallel analysis in addition to the eigenvalue >1 criterion. Finally, PCA identifies components rather than participant “clusters,” and the corresponding terminology should be corrected.

**********

what does this mean?). If published, this will include your full peer review and any attached files.

If you choose “no”, your identity will remain anonymous but your review may still be made public.

**Do you want your identity to be public for this peer review?**  For information about this choice, including consent withdrawal, please see our Privacy Policy

Reviewer #1: **Yes:**  Kaleem Arshad

Reviewer #2: No

Reviewer #3: No

**********

To ensure your figures meet our technical requirements, please review our figure guidelines: https://journals.plos.org/plosone/s/figures

You may also use PLOS’s free figure tool, NAAS, to help you prepare publication quality figures: https://journals.plos.org/plosone/s/figures#loc-tools-for-figure-preparation.

NAAS will assess whether your figures meet our technical requirements by comparing each figure against our figure specifications.

Attachment Submitted filename: Review-POLS one.pdf

# Author comment: Author response to Decision Letter 1

**DOI:** 10.1371/journal.pone.0360041.r002

8 Sep 2026

Dear editor and reviewers,

please find attached our point-by-point letter where we reply all your suggestions and comments.

Attachment Submitted filename: Response_to_reviewers_round_1.docx

# Peer review: Decision Letter 1

**DOI:** 10.1371/journal.pone.0360041.r003

**Authors:** Etsuro Ito

23 Sep 2026

Circulating low- and medium-molecular-weight adiponectin are associated with metabolic phenotypes in adults undergoing cardiometabolic screening

PONE-D-26-34597R1

Dear Dr. Sandoval,

We’re pleased to inform you that your manuscript has been judged scientifically suitable for publication and will be formally accepted for publication once it meets all outstanding technical requirements.

Within one week, you’ll receive an e-mail detailing the required amendments. When these have been addressed, you’ll receive a formal acceptance letter and your manuscript will be scheduled for publication.

An invoice will be generated when your article is formally accepted. Please note, if your institution has a publishing partnership with PLOS and your article meets the relevant criteria, all or part of your publication costs will be covered. Please make sure your user information is up-to-date by logging into Editorial Manager at Editorial Manager® and clicking the ‘Update My Information' link at the top of the page. For questions related to billing, please contact billing support.

If your institution or institutions have a press office, please notify them about your upcoming paper to help maximize its impact. If they’ll be preparing press materials, please inform our press team as soon as possible -- no later than 48 hours after receiving the formal acceptance. Your manuscript will remain under strict press embargo until 2 pm Eastern Time on the date of publication. For more information, please contact onepress@plos.org.

Kind regards,

Etsuro Ito, Ph.D.

Academic Editor

PLOS One

Reviewers' comments:

Reviewer's Responses to Questions

**Comments to the Author**

Reviewer #1: All comments have been addressed

Reviewer #3: All comments have been addressed

**********

2. Is the manuscript technically sound, and do the data support the conclusions?

Reviewer #1: Yes

Reviewer #3: Yes

**********

3. Has the statistical analysis been performed appropriately and rigorously?

Reviewer #1: Yes

Reviewer #3: Yes

**********

4. Have the authors made all data underlying the findings in their manuscript fully available?

The PLOS Data policy

Reviewer #1: Yes

Reviewer #3: Yes

**********

5. Is the manuscript presented in an intelligible fashion and written in standard English?

Reviewer #1: Yes

Reviewer #3: Yes

**********

Reviewer #1: I thank the authors for their detailed responses to my comments. Their revisions and clarifications adequately address the issues I raised:

Gel/membrane catalog number: Confirmed as a transcription error; corrected catalog number (4561086) and acrylamide gradient (4–15%) now provided.

RIPA buffer/non-denaturing conditions: The authors appropriately revised their terminology, acknowledging that strict native conditions cannot be fully claimed, and supported the protocol's validity with data from their own previously published work resolving adiponectin multimeric forms.

Serum input volume and voltage parameters: Rationale and optimization data (1–5 µL titration, ramped voltage protocol) were clearly provided, improving reproducibility.

Confirmation of transcription errors: Points 1 and 3 confirmed and corrected in the revised manuscript.

I am satisfied with the authors' responses and have no further concerns. I recommend the manuscript proceed, pending editorial discretion.

Reviewer #3: The manuscript has been properly revised. I have no further comments.I thought the English text was logical, easy to understand, and free of errors.

**********

what does this mean?). If published, this will include your full peer review and any attached files.

If you choose “no”, your identity will remain anonymous but your review may still be made public.

**Do you want your identity to be public for this peer review?**  For information about this choice, including consent withdrawal, please see our Privacy Policy

Reviewer #1: **Yes:**  Dr. Kaleem Arshad

Reviewer #3: No

**********

# Editor report: Acceptance letter

**DOI:** 10.1371/journal.pone.0360041.r004

**Authors:** Etsuro Ito

PONE-D-26-34597R1

PLOS One

Dear Dr. Sandoval,

I'm pleased to inform you that your manuscript has been deemed suitable for publication in PLOS One. Congratulations! Your manuscript is now being handed over to our production team.

At this stage, our production department will prepare your paper for publication. This includes ensuring the following:

* All references, tables, and figures are properly cited

* All relevant supporting information is included in the manuscript submission,

* There are no issues that prevent the paper from being properly typeset

You will receive further instructions from the production team, including instructions on how to review your proof when it is ready. Please keep in mind that we are working through a large volume of accepted articles, so please give us a few days to review your paper and let you know the next and final steps.

Lastly, if your institution or institutions have a press office, please let them know about your upcoming paper now to help maximize its impact. If they'll be preparing press materials, please inform our press team within the next 48 hours. Your manuscript will remain under strict press embargo until 2 pm Eastern Time on the date of publication. For more information, please contact onepress@plos.org.

You will receive an invoice from PLOS for your publication fee after your manuscript has reached the completed accept phase. If you receive an email requesting payment before acceptance or for any other service, this may be a phishing scheme. Learn how to identify phishing emails and protect your accounts at https://explore.plos.org/phishing.

If we can help with anything else, please email us at customercare@plos.org.

Thank you for submitting your work to PLOS One and supporting open access.

Kind regards,

PLOS One Editorial Office Staff

on behalf of

Prof. Etsuro Ito

Academic Editor

PLOS One
